# On the Size and Flight Diversity of Giant Pterosaurs, the Use of Birds as Pterosaur Analogues and Comments on Pterosaur Flightlessness

**DOI:** 10.1371/journal.pone.0013982

**Published:** 2010-11-15

**Authors:** Mark P. Witton, Michael B. Habib

**Affiliations:** 1 School of Earth and Environmental Sciences, University of Portsmouth, Portsmouth, United Kingdom; 2 Department of Sciences, Chatham University, Pittsburgh, Pennsylvania, United States of America; Ecole Normale Supérieure de Lyon, France

## Abstract

The size and flight mechanics of giant pterosaurs have received considerable research interest for the last century but are confused by conflicting interpretations of pterosaur biology and flight capabilities. Avian biomechanical parameters have often been applied to pterosaurs in such research but, due to considerable differences in avian and pterosaur anatomy, have lead to systematic errors interpreting pterosaur flight mechanics. Such assumptions have lead to assertions that giant pterosaurs were extremely lightweight to facilitate flight or, if more realistic masses are assumed, were flightless. Reappraisal of the proportions, scaling and morphology of giant pterosaur fossils suggests that bird and pterosaur wing structure, gross anatomy and launch kinematics are too different to be considered mechanically interchangeable. Conclusions assuming such interchangeability—including those indicating that giant pterosaurs were flightless—are found to be based on inaccurate and poorly supported assumptions of structural scaling and launch kinematics. Pterosaur bone strength and flap-gliding performance demonstrate that giant pterosaur anatomy was capable of generating sufficient lift and thrust for powered flight as well as resisting flight loading stresses. The retention of flight characteristics across giant pterosaur skeletons and their considerable robustness compared to similarly-massed terrestrial animals suggest that giant pterosaurs were not flightless. Moreover, the term ‘giant pterosaur’ includes at least two radically different forms with very distinct palaeoecological signatures and, accordingly, all but the most basic sweeping conclusions about giant pterosaur flight should be treated with caution. Reappraisal of giant pterosaur material also reveals that the size of the largest pterosaurs, previously suggested to have wingspans up to 13 m and masses up to 544 kg, have been overestimated. Scaling of fragmentary giant pterosaur remains have been misled by distorted fossils or used inappropriate scaling techniques, indicating that 10–11 m wingspans and masses of 200–250 kg are the most reliable upper estimates of known pterosaur size.

## Introduction

Comparisons between extinct animals and even highly derived modern descendants – as morphologically disparate as sauropod dinosaurs and birds, for instance - can provide a wealth of palaeobiological information about long dead forms. By contrast, students of groups with no modern descendents can only rely on close modern relatives to provide palaeobiological insights, and such comparisons are often considerably less informative. Not only may doubt exist over the relationships of the extinct group to modern animals, but their anatomy may be so different to that of extant forms that few meaningful insights can be drawn about their palaeobiology even if their taxonomic context is well understood. Both problems face researchers of pterosaurs, animals of controversial phylogenetic affinities [1]–[3] and very distinctive anatomy. Accordingly, pterosaur palaeontologists frequently rely on modern analogues rather than possible relatives for insights into pterosaur palaeobiology. Modern birds are commonly suggested to provide the best ecological and anatomical analogue and, by far, the most comparisons are made between pterosaurs and marine birds such as members of Laridae and Procellariiformes.

Pterosaur literature is rich with descriptions of pterosaurs flying and foraging in a marine bird-like manner (e.g. [4]–[8]). On occasion, the pterosaur-bird analogy has deepened to levels where some workers have applied ornithological terminology to pterosaur bones [9]–[10], analysed pterosaur anatomy for convergences with modern birds to deduce locomotory and ecological habits (e.g. [11]–[15]) or even treated pterosaurs and birds indistinguishably when estimating pterosaur masses [16]–[17].

Observations on avian flight have also heavily influenced research into pterosaur flight mechanics. It has commonly been assumed that pterosaurs and birds would take off in a similar way (e.g. [6], [17]–[20]) suggesting that pterosaurs leapt into the air with flapping wings or ran for a short duration to achieve the speeds necessary for flight. Studies into pterosaur flight suggest that the largest pterosaurs would struggle to take off with such a strategy, however, and many have concluded that giant pterosaurs required specific environmental conditions to launch and must be atypically lightweight to reduce the power required for flight.

Several recent studies have come to such conclusions. Chatterjee and Templin [19] insist that giant pterosaurs can fly, but only if they were extremely lightweight (16 kg for a 7 m span form; 70 kg for 10 m) and, ideally, employed downhill runs and headwinds when launching. They explicitly state that they see no method for launching a pterosaur above this mass ([19]; p. 19), despite contradictory evidence suggesting 25–50 kg and 200–250 kg are more realistic masses for 7 and 10 m span forms, respectively [21]–[25]. When discussing the feasibility of such low mass estimations, Paul [22] and Witton [24] noted that pterosaur bodies would require 60–90 per cent pneumaticity to reduce their masses to such levels; that ‘heavy’ pterosaur mass estimates are very comparable to the masses of modern birds and bats, and that low masses do not provide a sufficient quantity of soft tissue to cover pterosaur skeletons adequately. If these observations are accurate, Chatterjee and Templin's statements that a pterosaur massing more than 70 kg could not launch essentially renders any pterosaur above this size flightless. Using a wingspan/mass regression for the ‘heavy’ pterosaur dataset of Witton [24], this caps flighted pterosaur wingspans at 6.65 m.

A similar conclusion was reached by Sato *et al.* [17], who considered that birds – specifically ocean-going procellariiforms - and pterosaurs were so mechanically analogous that the flight mechanics of the former could provide insights into the flight of the latter. Regressing the masses of 7 and 10 m span pterosaurs from a procellariiform mass dataset, they predicted ‘heavy’ pterosaur masses and, by extrapolating flapping frequency against mass in albatross and petrels, suggested that a 5.1 m span and 41 kg mass was the pterosaur flight limit. They cast particular doubt on the flight abilities of *Pteranodon* and *Quetzalcoatlus northropi*, the largest representatives of two pterosaur clades that achieved gigantic size. According to Sato *et al.*, if these forms had narrow, albatross-like wings, they would be incapable of flight in modern environments. While they acknowledge not factoring flight strategies such as thermal soaring into their considerations, Sato *et al.* conclude that the largest pterosaurs were probably flightless without constant, strong winds or different atmospheric and gravitational conditions to those in modern times.

Most recently, Henderson [25] suggested that *Q. northropi* was completely incapable of flight due to its mass, which he predicted to be 544 kg. It was stated that assuming flightlessness for this taxon “…frees us from the mental gymnastics required to generate an anatomy with sufficient muscle mass and power to be able to fly when possibly weighing more than thirty times that of the heaviest, living, volant birds such as the 16-kg Kori Bustard (*Ardeotis kori*) and the Great Bustard (*Otis tarda*), which may attain 22 kg in some cases. These birds seem to be at the upper mass limit for flying given their apparent difficulty in taking off.” (p. 783). A 22 kg pterosaur would equate to a 4.2 m wingspan (using the wingspan/mass regression of Witton [24]) and suggest a considerable number of pterosaur taxa had far outgrown the limits of flight. Direct evidence for this in *Q. northropi* is suggested by its allegedly short wings and, apparently mirroring the condition in modern flightless birds, by being considerably larger than its volant counterparts [25].

All three of the studies discussed above share common comparisons between pterosaurs and birds and, here, we argue that these authors have relied too heavily on this analogy. Moreover, their conclusions do not seem to have shown much consideration of other evidence for the flighted nature of pterosaur giants. Subsequently, we attempt to review several aspects of giant pterosaur palaeobiology that provide insights into their flighted or flightless nature. Chiefly, we model the flight of the 10–11 m span *Quetzalcoatlus*, one of the largest azhdarchid pterosaurs and flying animals known. In preparation for this analysis, we reappraised the wingspan and mass estimates of giant azhdarchids to determine whether their massiveness alone will render them flightless [25] and ensure our flight study uses the most reliable parameters of known azhdarchid size possible at the time of writing. In addition, the bone mechanics, scaling regimes and flight kinematics of birds and pterosaurs are compared to assess their mechanical interchangeability, and we review the anatomy associated with flight and terrestrial locomotion in giant azhdarchids and the largest ornithocheiroid, the 7 m span *Pteranodon*. Evidence for and against flighted lifestyles from their sedimentological contexts of these pterosaurs is also presented, and we compare the probable flight styles of the largest pterosaurs, demonstrating that different giant taxa employed distinct flight styles and cannot be treated as mechanically analogous as they have been in some studies.

(Note that while many pterosaurs were large, we restrict our use of the term ‘giant’ to *Pteranodon* and the largest azhdarchids, pterosaurs that achieved the maximum sizes of the two major pterodactyloid bauplans: narrow-winged, small-bodied ornithiocheiroids and larger bodied lophocratians (see [26]). Non-pterodactyloid pterosaurs do not appear to have exceeded wingspans of 3 m [27] and most pterodactyloid groups contain taxa attaining 4–6 m wingspans [6], [8]. By contrast, the largest pteranodontids are known to have achieved wingspans of around 7 m [12] and the largest azhdarchids are estimated to span at least 10 m ([28], but also see below). Our focus on giant taxa does not preclude the loss of flight in smaller pterosaurs, but does provide insight into whether size alone is a limiting factor on pterosaur flight ability.)

## Methods

### Giant pterosaur size estimation: wingspans

Accurately modelling the size of giant forms is essential to appreciating their flight ability as even relatively small over-predictions of wingspans may translate to considerable over-estimates of mass and subsequently inaccurate appreciation of flight performance. The maximum size estimates for *Pteranodon* are well constrained by relatively complete specimens of giant individuals [12], but the largest azhdarchids are represented by extremely fragmentary material that make estimating their size problematic. Of the three named giant azhdarchids, *Q. northropi* is only known from a complete, 544 mm long left humerus and other fragmentary components of the same wing [29]; *Arambourgiania* from an incomplete (?fifth) cervical vertebra and some referred bone fragments [9]–[10] and *Haztegopteryx* from a weathered, broken proximal left humerus, fragmentary cranial material and a referred, incomplete left femur [30]–[31]. Accordingly, reconstructing the size of these pterosaurs has required significant extrapolation from smaller azharchids such as the 5 m span *Quetzalcoatlus* sp. and 3.5 m span *Zhejiangopterus* [28], [32]–[33] and has resulted in wingspan estimates ranging from 10–13 m (e.g. [9], [19], [30]). Though only a difference of 30 per cent, pterosaur scaling coefficients (e.g. [19], [24]) predict that a 13 m span pterosaur will mass almost twice that of a 10 m span individual, stressing the importance of accurately assessing the wingspans of these forms.

With this in mind, we have reappraised the size estimates of giant azhdarchids to ensure that the data used in our flight calculations are as accurate as currently possible. The data used in size estimates of *Q. norhtropi* have not been published, but an approximate wingspan of 10–11 m has been verified by one of the authors (MBH) and independent researchers with access to the *Quetzalcoatlus* sp. material (e.g. Cunningham and Bennett, pers. comm. 2009). These estimates are in agreement with *Q. northropi* size predictions based on published datasets of azhdarchid wing proportions (e.g. [34]). We are confident, therefore, that the wingspan of *Q. northropi* has been as well modelled as can be expected given the available evidence, but the same cannot be said for other giant azhdarchids. The size of the holotype *Arambourgiania* individual has been estimated twice using data from *Quetzalcoatlus* sp. [9], [35] and, in each case, a wingspan of 11–13 m was predicted. Both estimates, however, isometrically scaled the bones of smaller azhdarchids until they attained cervical vertebra metrics comparable with those of *Arambourgiania*, a method that ignores Wellnhofer's [36] observations that pterosaur necks grow with positive allometry against body size. Such allometry in neck length is known in a suite of other long-necked animals including giraffes, sauropod dinosaurs [37]; protosaurs [38] and plesiosaurs [39]. It is likely, therefore, that azhdarchid necks demonstrated similar allometry. If so, the 5 m span forms used in predicting an 11–13 m wingspan for *Arambourgiania* would have relatively short necks and, when scaled isometrically to fit the neck of *Arambourgianaia*, will over-estimate its wingspan.

Unfortunately, we still lack sufficient azhdarchid remains to permit a study into the growth allometry of azhdarchid necks and a more confident wingspan prediction cannot be made at present. Company *et al.* (in Pereda Suberbiola *et al.* [40]) stated that a wingspan of 7 m was likely for *Arambourgiania* but did not provide any rationale for their estimate. Given that the incomplete *Arambourgiania* holotype vertebra is approaching a metre in length, we suggest their estimate is probably too low and that *Arambourgiania* was comparable in size to *Q. northropi*. Of course, such speculations are of no use in mathematical modelling of pterosaur flight and until further data on the allometry of azhdarchid necks or additional remains of *Arambourgiania* are presented, we refrain from including size estimates of *Arambourgiania* in our analysis.

**Figure 1:**
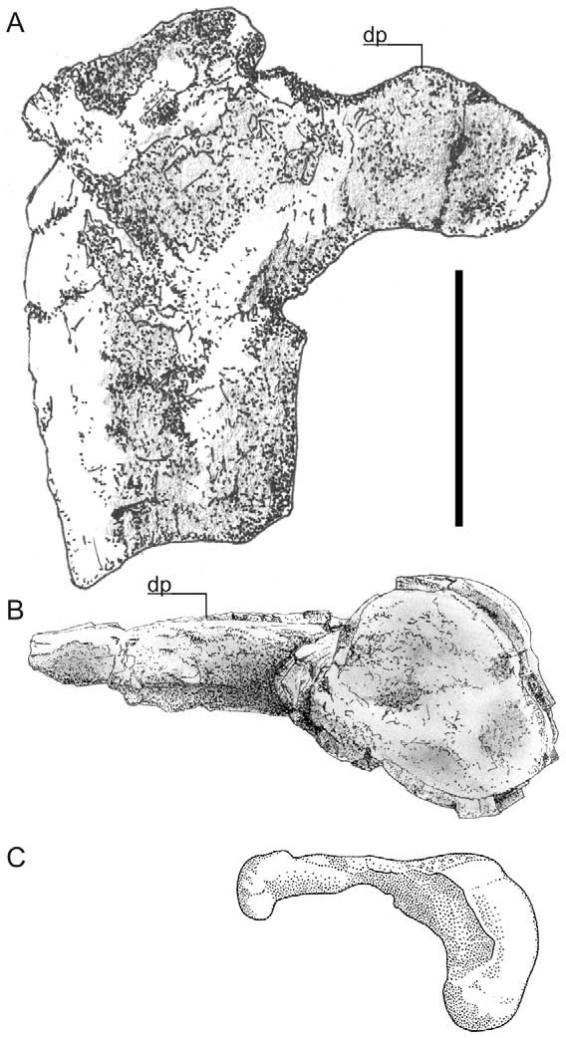
Azhdarchid humeri. A, left *Hatzegopteryx* humerus in ventral view; B, distal view; C, right *Quetzalcoatlus* sp. humerus in proximal view. Note the distorted diaphysis of the *Hatzegopteryx* humerus compared to the undistorted profile of *Quetzalcoatlus* sp.. C, from Padian and Smith [77]. Scale bar represents 100 mm.

The most recent giant pterosaur to be described and named, *Hatzegopteryx*, was proposed to have a wingspan between 10–12 m based on its marginally wider humeral diaphysis than that of *Q. northropi* (90 *vs.* 80 mm, respectively; [30]–[31]). This suggested that the humerus had to be somewhat longer than that of *Q. northropi* and, accordingly, indicated a larger wingspan. These findings were noted to be somewhat paradoxical, as the proportions of the proximal humerus were very similar to those of *Q. northropi* [30]–[31]. Reappraisal of this material reveals the details of this paradox: the *Hatzegopteryx* humerus has undergone post-depositional distortion that has dorsally deflected the deltopectoral crest so that, rather projecting anteriorly, it projects anterodorsally (Fig. 1). As such, the 90 mm figure reported as the anteroposterior dimension actually measures the diaphysis posteroventrally – anterodorsally. When the distortion of the humerus is corrected for, the actual anteroposterior diaphysis dimension measures 80 mm, a figure matching that of *Q. northropi* and an indication that the two taxa were of very similar size. With the 11–13 m wingspan of *Arambourigania* also unlikely, we conclude that there is presently no evidence for pterosaurs with wingspans beyond 10–11 m. Accordingly, this has been taken as the largest reliable size record for any currently known species and is used in the following investigations into pterosaur flight mechanics.

### Giant pterosaur size estimation: mass

Even with constrained wingspans, opinions on giant pterosaur masses are extremely controversial with fivefold differences in mass estimations existing for some taxa [24] The half-tonne azhdarchid recently proposed by Henderson [25] makes previous ‘heavy’ estimates look positively lean in comparison, being a predicted mass almost double that of any previously published figure. The estimate was generated through a mathematical 3-dimensional slicing model of *Q. northropi* and, if accurate, Henderson is correct in suggesting it will almost certainly be too heavy to fly.

Slicing methodology itself has no obvious flaws, but its accuracy is dependent upon the three dimensional digital sculpture sliced by the algorithms. The model of *Q. northropi* used by Henderson [25] had a torso 1.8 meters in length and was apparently based on proportionally incorrect reconstructions in semi-technical literature [6], [28]. Examination of more complete *Quetzalcoatlus* sp. material (Habib, unpublished data; Langston, pers comm.) indicates his reconstructed torso is too large by a factor of about 2.77 (actual thorax and abdomen total length is predicted at approximately 0.65 meters for *Q. northropi*). Note that such a body length is not only predictable using *Q.* sp.: the Henderson *Q. northropi* has body proportions quite unlike that of any pterosaur, including the others modelled with far more precision in the same study. Utilizing a more appropriate body length estimate, the mass estimate for the Henderson [25] *Quetzalcoatlus* model drops from 544 kg to about 240 kg, which is congruent with estimates by Paul [22] and Witton [24], and is comfortably within the possible range of pterosaur flight predicted by Habib [41].

Henderson [25] produced an alternate model of *Quetzalcoatlus* which had widths and depths of the torso reduced by 20% and 25%, respectively. While the shape of this model is still inaccurate, the volume turns out to be somewhat closer to the actual volume estimated by scaling *Quetzalcoatlus* sp. The axial mass of the alternate model had a mass of 198 kg (compared to 474 kg in the original), which begins to approach the estimates of other recent works - but Henderson [25] rejects this model as having insufficient force-producing cross-sectional area to enable take-off and active flight. While we note that no analysis was provided to support this assertion, this slender model has been constructed arbitrarily and will not be considered further here.

Henderson also provided an additional method of predicting the mass of a giant azhdarchid by utilizing simple geometric scaling of *Tupuxuara* to *Q. northropi* proportions. This resulted in a mass of 461 kg, but the scaling estimate was performed using a wingspan of 11.2 meters for *Quetzalcoatlus*, a likely overestimate (see above) and relies on geometric similarity between *Tupuxuara* and azhdarchids. These forms differ quite considerably in some proportions (particularly around the head and neck) and more applicable scaling of *Q. northropi* from other, completely known azhdarchids produces a much lower mass estimate. Accordingly, while we salute the thoroughness and detail of Henderson's work (and find the mass estimates of his other pterosaurs reasonable), we find his predictions of giant azhdarchid mass to be flawed and a poor rationale for grounding giant pterosaurs. Accordingly, for reasons outlined by Paul [21]–[22]; Witton [24] and (in part) Henderson [25], we prefer traditionally ‘heavier’ pterosaur mass estimates and use them in our study here (e.g. 180–250 kg for *Q. northropi*) and do not consider ultra-light or ultra-heavy pterosaur masses to be well rationalised.

### Bone strength analysis

Analysis of limb bone strength in bending can be used in a comparative context to determine if giant pterosaur skeletal spars could sustain anticipated flight forces, and to determine if pterosaur launch and flight dynamics were likely to have been similar to that of living birds. Estimates of bone strength in pterosaur long bones (and cervical vertebrae, for *Quetzalcoatlus* sp.) were derived from applying a beam model and calculating section modulus at the nearly elliptical midshaft as per the methodology of Habib [41]. Bending and torsional loads predominate in vertebrate limb bones [42]–[47] and we accordingly evaluated structural characteristics related to bending and torsional strength, taking bone strength as inversely related to maximum stress under loading. Using a beam model, maximum stress in bending is given by My/I (where M is the bending moment, I is the second moment of area about the neutral axis, and y is the maximum distance from the neutral axis to the edge of the section) [48]. The section modulus, Z, in bending is defined as I/y, and in torsion as J/r. M and T can be reasonably considered to be proportional to the product of body mass (B) and bone length (L) (femoral or humeral) [49]–[51]. Therefore, bone structural strength ∝ Z/(B*L). The polar section modulus (Zp) is related to both torsional and (twice) average bending strength in any two perpendicular planes [52], and is the measure used for estimating strength in this study. A more complete examination of these methods and their results, applied to living avian taxa, can be found in Habib & Ruff [53] and for both birds and pterosaurs in Habib [41].

To determine the relative failure load (failure force in body weights) of a bone, an estimate of material load capacity for pterosaur bone is required. Kirkpatrick [54] provides experimental values for load capacity of both avian and chiropteran bone. While both are vertebrate flyers, bats are highly apomorphic in having exceptionally compliant bone [55]. Birds are phylogenetically closer to pterosaurs, and given that we wish to compare the mechanics of avian bone and pterosaur bone, this is a more useful estimate and we apply Kirkpatrick's result of 175 MPa for the breaking limit of bird bone as the limit for pterosaur bone. However, this is a conservative estimate, as Kirkpatrick's result falls below the failure stress recorded for most other vertebrate bone, including those for avian hindlimb bones and most mammalian long bones [56].

Exact sections, as derived from CT imaging, were not available for the pterosaur species examined in this study (except for *Bennettazhia*). However, measurements of external breadths and cortical thickness were taken manually from pterosaur long bones at the Smithsonian's National Museum of Natural History (NMNH) in Washington, DC, the Texas Memorial Museum (TMM) in Austin, TX, and the Bavarian State Palaeontological Collection (BSPG) in Munich, Germany (cortical breadth was measured from broken elements). External and internal measurements were also available in the literature for *Montanazhdarcho* [57] and measured by CT imaging in the case of the humerus of *Bennettazhia oregonensi*. These measurements indicate that the midshaft of the humerus and femur of most pterosaurs closely approach a true ellipse. Modelling the midshaft as a true ellipse yields a simple formula for the calculation of Zp:
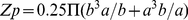
(1)Where ‘a’ and ‘b’ are the radii of the ellipse in any two perpendicular planes. For this study, ‘a’ and ‘b’ were taken as the dorsoventral and anteroposterior directions, respectively. This formula is exact only for symmetric sections, but it is a strong approximation when the section closely approaches perfect symmetry, which all of the measured pterosaur elements do at their midshaft (the measured location for each bone). The above formula, as written, gives the section modulus for a solid section. To calculate the value of Zp for a hollow section, the polar second moment of area (J) was calculated for both the outer and inner diameters using:
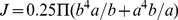
(2)The inner diameter value (medullary cavity J) is then subtracted from the total, solid section value of J. We then calculated the final value of Zp as cortical J/average radius of section (in the x and y planes). We report the results from the azhdarchid sample, specifically, which is most relevant to the current consideration of gigantism in pterosaurs.

### Flap gliding performance

Basic estimates of soaring and short-term flapping potential in giant pterosaurs were calculated using the same general approach suggested by Pennycuick [58] for the estimation of flapping frequency and glide performance of birds. Glide performance for a static planform and weight is easily calculated, but flying animals are more complex due to their ability to adjust span to change wing loading during glides and, during long transits, reduce weight as fat reserves are consumed. This makes an iterative formulation more applicable. We utilized the latest version of Colin Pennycuick's *Flight* program to make our flight calculations. The software is freely available from http://www.bio.bristol.ac.uk/people/pennycuick.htm and allows a wide range of input parameters. The program also includes a wide range of data from measured, living birds (both wild and captive specimens) although the program parameters need to be altered from their defaults to account for pterosaur biology.

Membrane wings are able to provide higher maximum lift coefficients than avian wings, and the membranous wings of bats are expected to have a steeper lift slope than the stiffer, less compliant wings of birds [59]. The work by Song *et al.* [59] indicates that compliant, membrane wings achieve greater maximum lift coefficients than rigid wings, but data have yet to be collected demonstrating that this holds in vivo for bats and birds. We assume that the same generalities apply to pterosaur wings, though their wing structure, and specifically membrane histology, is somewhat different from those seen in living fliers [e.g. 60]–[62]. The maximum lift coefficient for most pterosaur wings is expected to have exceeded that measured for birds, by about 33% (Cunningham, pers com.) and was set at 2.2 for the pterosaur analysis, and may have therefore been as high as 2.0 to 2.2 under unsteady conditions. We set a maximum steady coefficient of 1.8, which is the absolute maximum measured for birds under steady state conditions [58], but should not have been extraordinary for pterosaurs. For large pterosaurs, the ratio of flap-powered flight to gliding cycles would have been quite short, which is accounted for by setting a flap∶glide ratio of 0.2 in the Flight program. Finally, the membrane wings of pterosaurs would have been incapable of producing useful fluid forces at the extreme span reductions sometimes used by birds (due to aeroelasticity of the wing membrane and the tendency to flutter when slack): span reduction was subsequently limited to a hard stop at 80% of the resting span by setting the Bstop value to its maximum, which means that span is kept near maximum throughout flight (in this case, Bstop = 6, which means that span is 80% of full span at double the stall speed). Note that these are still rough estimates; because pterosaurs cannot be reliably treated as birds, we can only utilize the aforementioned algorithms for those parameters that are widely applicable across flying animals in general (that is, those factors extracted from first principles). Of the calculated parameters, the best glide speed is the most robust, because it varies independently of physiology: the best glide speed depends on planform, mass, and wing efficiency, and the parameters are therefore not specific to birds and can be accurately extrapolated to pterosaurs so long as accurate estimates of wing shape, body size, frontal area, and membrane lift coefficient are utilized (the same calculations can even be reasonably applied to sailplanes and other human-constructed devices).

In order to complete estimates of flight performance for any animal, information on the wing shape and size (planform) is required. Because pterosaur wing shape is not known with precision, we ran calculations for several different possible configurations, and report the estimates of wing area given by various authors utilizing different wing shape reconstructions.

## Results

### Flap gliding analysis

**Figure 2:**
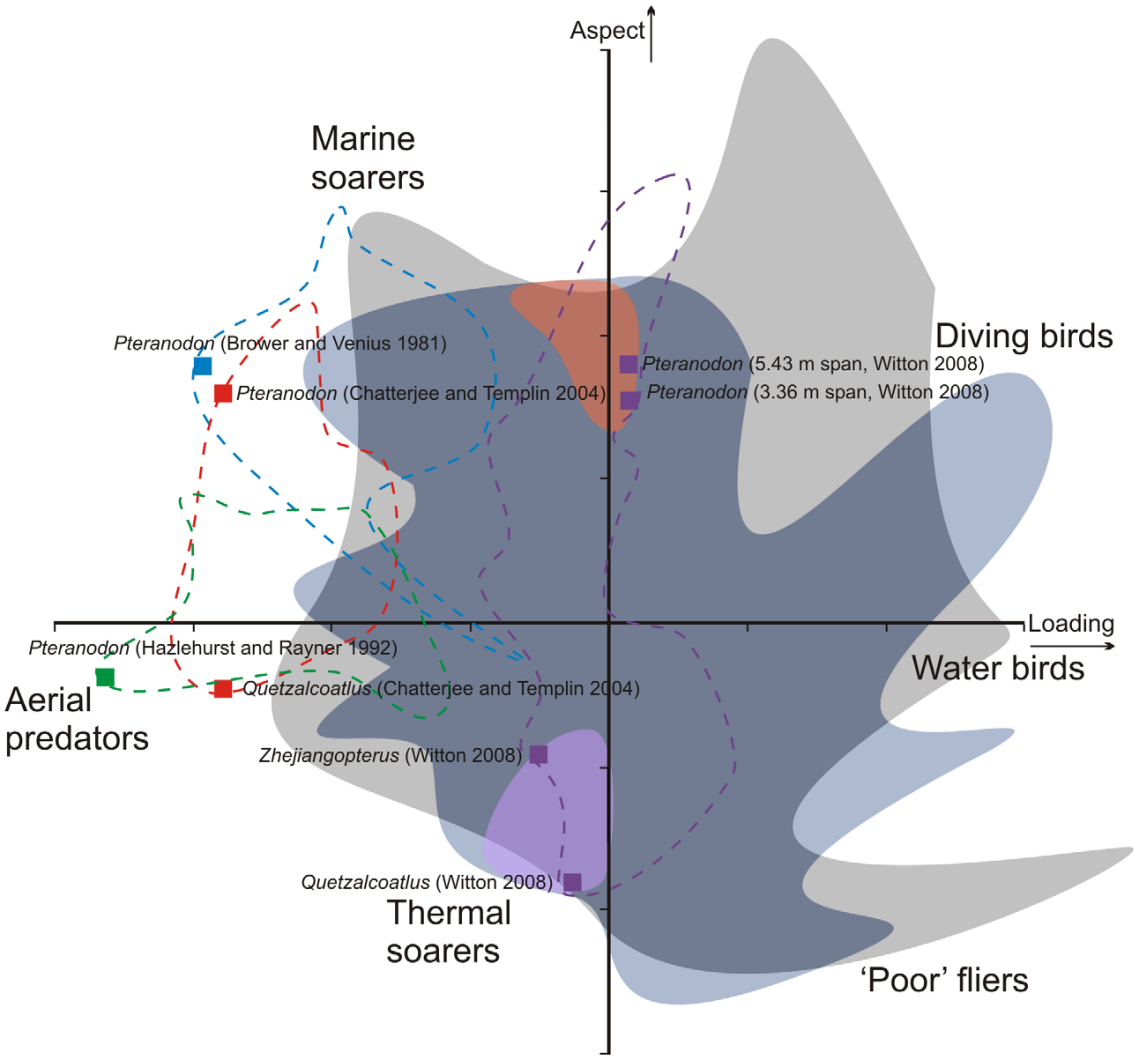
Pterosaur and soaring bird wing ecomorphospace compared using principal component analyses from Norberg and Rayner [**63**] and Rayner [**64**]. Blue shading, wing ecomorphospace of modern birds (from [64]); grey shading, modern bats (from [63]); orange shading; dynamically-soaring birds (tropic birds, petrels, albatrosses); purple shading, statically-soaring birds (condors, vultures, storks; cranes); purple dashed line, extent of pterosaur wing ecomorphology found in Witton [24]; blue dashed line, pterosaur wing ecomorphology of Brower and Venius [81]; green dashed line, broad-winged pterosaur wing ecomorphology of Hazlehurst and Rayner [11]; red dashed line, pterosaur wing ecomorphology of Chatterjee and Templin [19].

Recent reconstructions of body mass and planform for the pterosaurs *Pteranodon* and *Quetzalcoatlus*, along with prior estimates from the literature, are presented in Table 1. The likely wing loading and aspect ratio reconstructed for *Pteranodon*, according to the methodology of Witton [24] are roughly similar to that of long-winged seabirds, including procellariiform seabirds, and are consistent with a rapid flap-soaring flight dynamic over open ocean environments. However, the reconstruction of *Quetzalcoatlus* produced by the quantitative metric of Witton [24] generates an expected planform outside the range of shapes previously measured for living long-winged seabirds (Table 1). Pterosaur and soaring bird wing ecomorphospace comparisons, using principal component analyses and incorporating data from Norberg and Rayner [63] and Rayner [64], indicate that most pterosaurs are incomparable to soaring procellariiform birds (Fig. 2) and particularly so when ‘heavy’ pterosaur mass estimates are utilized. The dynamic soaring utilized by living Procellariiformes requires high aspect ratio, high efficiency wings and a high wing loading to promote rapid glide speed [65]. Some narrow-winged, heavily loaded pterosaurs (principally ornithocheiroids) overlap with the ecomorphospace of albatross and similar dynamic soaring birds but most pterosaurs fall outside of this shape space, including the giant azhdarchids.

**Table 1:** Wing attributes modelled for giant pterosaurs and procellariiforms.

Taxon	Common name	Wingspan (m) *b*	Mass (kg) *M*	Wing area (m^2^) *S*	Weight (Mg) *N*	Aspect ratio (*b* ^2^/*S*)	Wing loading (*N*/*S*)	Reference
**Pterosaurs**								
*Pteranodon*	-	6.95	14.95	2.53	146.56	19.10	57.93	[81]
*Pteranodon*	-	6.95	16.6	4.62	162.85	10.455	35.248	[11]
*Pteranodon*	-	6.95	16.60	2.65	162.85	18.23	61.45	[19]
*Pteranodon*	-	5.43	36.68	1.60	359.84	18.42	224.79	[24]
*Quetzalcoatlus*	-	10.39	70.00	9.55	686.70	11.30	71.91	[19]
*Quetzalcoatlus*	-	9.64	259.06	11.36	2541.40	8.18	223.66	[24]
**Birds**								
*Diomedea exulans*	Wandering albatross	3.46	8.16	0.66	80.05	18.00	120.71	[122]
*Diomedea exulans*	Wandering albatross	3.03	8.73	0.61	85.64	15.03	140.17	[101]
*Diomedea irrorata*	Waved Albatross	2.31	2.04	0.36	20.01	15.00	56.11	[122]
*Thalassarche melanophrys*	Black-bowed albatross	2.16	3.79	0.36	37.18	13.11	104.44	[101]
*Thalassarche chrysostoma*	Grey-headed albatross	2.18	3.79	0.35	37.18	13.50	105.62	[101]
*Phoebetria* sp.	Sooty albatross	2.18	2.84	0.34	27.86	14.06	82.43	[101]
*Macronectes* sp.	Giant petrel	1.99	5.19	0.33	50.91	11.96	153.82	[101]
*Procellaria aequinoctialis*	White-chinned petrel	1.40	1.37	0.17	13.44	11.60	79.52	[101]
*Fulmarus* sp.	Fulmar	1.13	0.82	0.12	8.00	10.30	64.48	[101]
*Puffinus pacificus*	Wedge-tailed shearwater	1.01	0.38	0.10	3.73	10.20	37.28	[123]
*Puffinus nativitatis*	Christmas shearwater	0.82	0.34	0.07	3.34	9.61	47.65	[123]

Flap-gliding performance analysis using the altered equations from Pennycuick [58] also provide a solution to the problem of long distance travel in giant pterosaurs, which otherwise would seem to be above the size limits for sustained flapping flight. For *Quetzalcoatlus*, using the narrow planform of Chatterjee and Templin [19], the estimated best glide speed is 13.3 m/s, and the speed for minimum sink rate is 8.80 m/s. If *Quetzalcoatlus* was able to work under anaerobic power (see below) to climb out for one minute after launch, this minimum sink speed would provide over a half kilometre of range to reach an external source of lift. However, the situation is more favourable with heavier body masses because it provides substantially more total muscle power and much greater glide speed once the animal begins soaring. Under the broader wing shape of Witton [24], the expected best glide speed for *Quetzalcoatlus* is 24.9 m/s, and the minimum sink speed is 16.3 m/s. The minimum sink speed would therefore provide close to a kilometre of distance under a one-minute burst, minus distance lost to climbout altitude gain. However, most soaring animals today fly at their minimum sink speed when using thermal soaring and certain forms of shear lift [58], [66]. The maximum range speed may be a more reasonable estimate of the climbout velocity, especially for an animal trying to reach external lift sources. Assuming that *Quetzalcoatlus* carried mostly anaerobic muscle in its flight muscle mass, as predicted by Marden [23], and using the maximum power output of anaerobic avian muscle ([67] - a conservative estimate, as other diapsids produce more relative power from anaerobic muscle), the expected maximum range speed under the Witton [24] morphology is 48.3 m/s with a climbout altitude gain of 1 m/s. Taken alone, these figures indicate a one-minute burst range of 2.88 km. Of course, considerable time and power would be required to accelerate to the extremely high maximum range speed, but even with those considerations, the range to external lift under an aerobic burst would likely exceed 1.5 km.

### Bone strength analysis

Pterosaur humeri are consistently stronger than expected that would be expected from avian structural scaling (Table 2). Relative Failure Force (RFF) gives the ratio of force required for failure in simple bending for cantilever style loading (total length = moment arm) divided by the body weight of the animal. Note that in life, very few long bones actually load as cantilevers; the actual moment arm length is usually much shorter, but can be taken as proportional to total element length. Therefore, the RFF produces a size-corrected comparison value that can be used to assess the relative structure robustness of elements across taxa. The avian expectation is the predicted bone strength for a bird with the same mass as the pterosaur listed in each row. Compared to the avian expectation, the measured pterosaur humeri are universally robust: the closest any come to an avian-strength humerus is *Montanazhdarcho*, which we estimate to have a RFF 1.9 times that of an average bird at the same mass. The RFF values for *Quetzalcoatlus northropi*, calculated at three different possible body masses (180 kg, 200 kg, and 250 kg; Table 2) and were always found to have RFF more than twice the expected value for a bird scaled to the same size; in the case of the 180 kg reconstruction, the RFF approaches triple the avian expectation (ratio of 2.82). Interestingly, with regards to the proximal forelimb, this means that the gap between pterosaur and avian structural strength is largest at the greatest body masses. Bird humeri scale near isometry, showing very weak negative allometry [41]. Pterosaur humeri are relatively more robust at large sizes, indicating a trend of positive allometry. By comparison, the femur of *Quetzalcoatlus* is quite gracile, with a RFF below one (meaning it would fail in pure cantilever bending), which is less than a third of the value for an average bird femur at the same body mass. Note that the fifth cervical vertebrae of *Quetzalcoatlus* sp. - modelled as a beam because of its unusual tubular, elongate shape - is actually twice as strong in cantilever bending as either femur in the same animal.

**Table 2:** Bending strength of several major bones in *Quetzalcoatlus* and two other azhdarchid pterosaurs.

ID	Taxon	Element	Section Modulus (length corrected)	Wingspan (m)	Body Mass (kg)	Relative Failure Force	Avian Expectation	Observed Failure Force/Avian Expectation
USNM 11925	*Bennettazhia oregonensis*	Humerus	3.43	2.80	6.10	7.16	2.69	2.66
MOR 691	*Montanazhdarcho minor*	Humerus	2.75	3.00	7.26	4.84	2.55	1.90
TMM 41961	*Quetzalcoatlus* sp.	Humerus	8.37	4.70	22.34	4.78	1.78	2.69
TMM 41961	*Quetzalcoatlus* sp.	Cervical 5	3.51	4.70	22.34	2.00	n/a	
TMM 41961	*Quetzalcoatlus* sp.	Femur	1.66	4.70	22.34	0.95	3.15	0.30
TMM 41450	*Quetzalcoatlus northropi*	Humerus	36.18	10.40	180.00	2.56	0.91	2.82
TMM 41450	*Quetzalcoatlus northropi*	Humerus	36.18	10.40	200.00	2.31	0.88	2.62
TMM 41450	*Quetzalcoatlus northropi*	Humerus	36.18	10.40	250.00	1.85	0.82	2.25

The ‘avian expectation’ indicates the expected strength in bending for each element if pterosaurs followed the structural scaling of birds. Relative Failure Force is the ratio of cantilever failure force, in bending, divided by body weight. Note that the proximal forelimb of the sampled pterosaurs is much stronger than expected from the avian trend, but that the femur of *Quetzalcoatlus* is only 30% as strong in bending as would be expected for a bird of its size.

## Discussion

### Were giant pterosaurs flightless?

#### Bone mechanics

**Figure 3:**
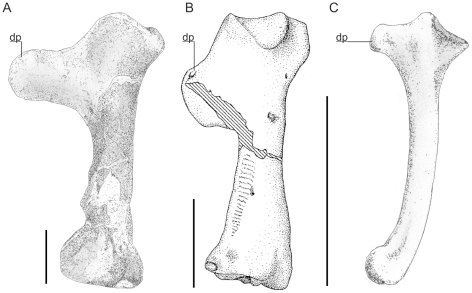
Dorsal views of giant and tiny pterosaur humeri. A, *Quetzalcoatlus northropi* (10–11 m wingspan); B, *Pteranodon* (7 m wingspan); C, *Pterodactylus* (45 cm wingspan). Note that each bears a large deltopectoral crest (dp) and robust extremities. Scale bars represent 100 mm (A and B) and 10 mm (C). A and C, from Witton *et al.* [121]; B, modified from Bennett [12].

**Figure 4:**
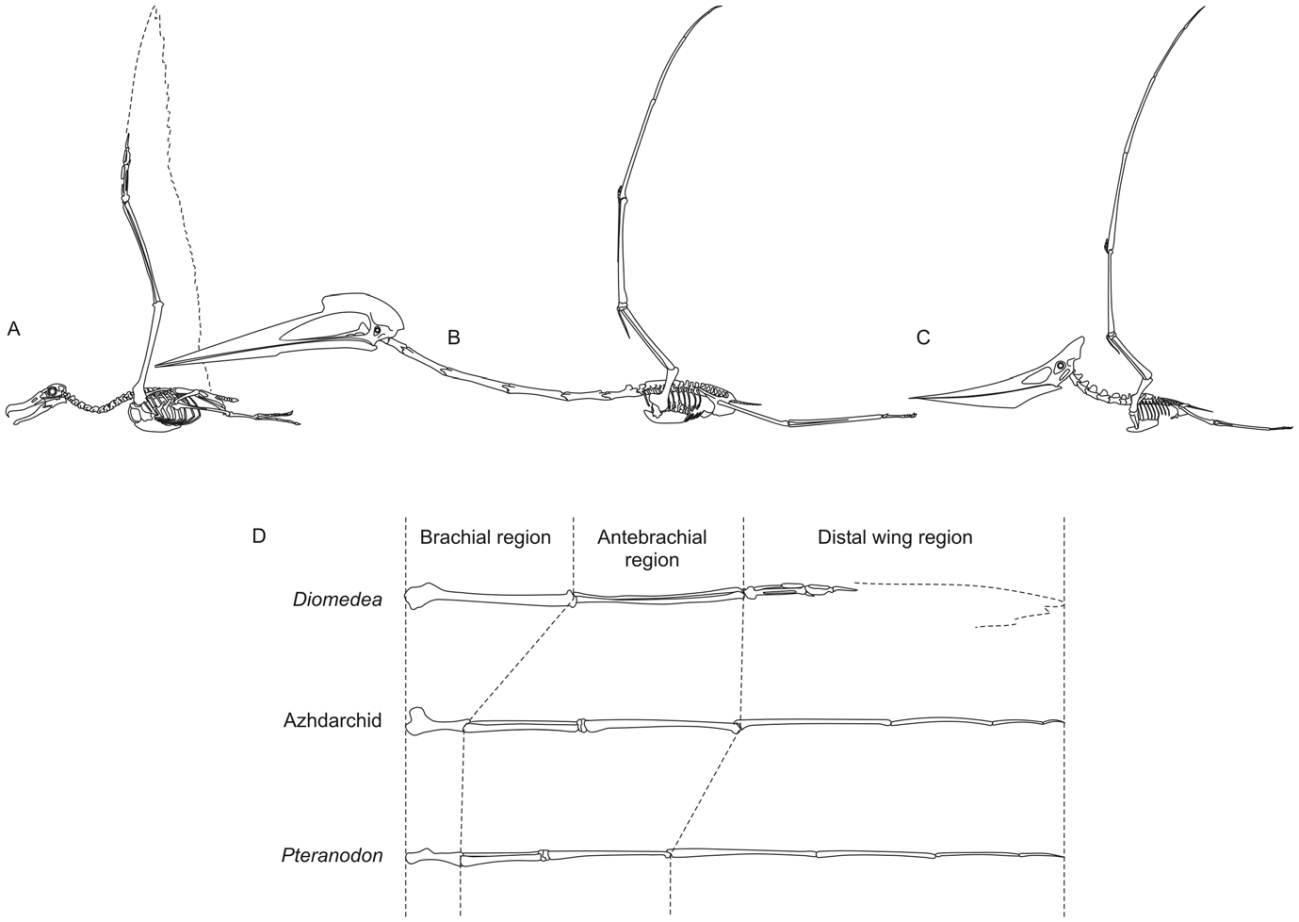
Albatross, azhdarchid and pteranodontian skeletons compared. A, wandering albatross, *Diomedea exulans*; B, the azhdarchid *Hatzegopteryx*; C, the pteranodontian *Pteranodon*; D, functional wing region dimensions compared across a standard wing length. A, based on Paul [22]; B, based on Buffetaut *et al.* [30]–[31], Kellner and Langston [33], Cai and Wei [32] and Pereda Suberbiola [40]; C, based on Bennett [12]; D, functional regions taken from Prondvai and Hone [111]. Images not to scale.

There are several potential lines of enquiry to assess flightlessness in pterosaurs: their bone structure, flight adaptations, terrestrial competence and depositional context. The investigation into bone strength carried out here sheds light on the first of these points, demonstrating that at least some aspects of pterosaur skeletons are far more robust than expected for animals of their size. Although many of their bones were hollow [15], [68], pterosaur skeletons should not be considered delicate or fragile in the typical sense: our bone strength analysis shows that pterosaur humeri are up to three times more resistant to failure than those of birds thanks to their diaphyses expanding at a much greater rate with increasing body size (Fig. 3). Actual failure loads estimated from cross-sectional properties indicate that pterosaur humeri were more than strong enough to sustain flapping loads. Other bones of the pterosaur wing are also proportionally more robust in larger forms, indicating the entire wing skeleton shared the same increased resistance to mechanical failure. This trend is the reverse of that seen in birds where the bones become relatively slender with increasing size (Fig. 4). Consequently, average avian trends in skeletal strength [41] suggest that a bird of equivalent size to *Quetzalcoatlus northropi* must be flightless as the expected cantilever failure force per wing would be less than one body weight and therefore the wing would fail during flight (Table 2). In addition, although bird femora are proportionally stronger than those of pterosaurs, this does not mean that pterosaur femora were mechanically weak. Habib [41] found that birds massing over 500 g show strong positive allometry in femur strength and Prange *et al.* [69] demonstrated that bird femora have much greater proportionality coefficients than those of mammals. Bird femora are therefore simply bigger than predicted for their body mass (see discussion below), whereas those of pterosaurs are in keeping with their body size. In addition, other authors have commented that pterosaur femora only appear slender in comparison to their large forelimbs and were well suited for powerful leaping [18], [70].

The structure and scaling properties of giant pterosaur bones are confusing if giant pterosaurs were flightless. If giant pterosaurs had abandoned flight it may be predicted that their bone strengths would correlate well with those of comparably-sized terrestrial animals, but they appear considerably over engineered by comparison. Although mammal humeri are unpneumatised, bone structural strength is primarily influenced by bone diameter: thus, while pterosaur bone loses some strength by being hollow, it is stronger against torsion and buckling than a solid bone of comparable length and mass. The importance of diameter in bone strength means that the diameters of pterosaur humeri can be roughly compared with those of terrestrial mammals. Among terrestrial mammals, humeral proportions akin to those of giant azhdarchids are only seen in the largest, heaviest forms such as giraffes and hippopotamuses [71], animals with masses considerably higher than even the largest pterosaurs (e.g. 2.4 tonnes in *Hippopotamus* [72]). It can be seen, therefore, that pterosaur humeri scale with much greater allometry than is necessary for a terrestrial animal and would have considerably higher RFF values than those of comparably sized mammals. In all, we find it difficult to explain why pterosaur limbs were of such considerable strength if they were not subjecting their skeletons to high mechanical stresses such as those experienced during flight.

#### Flight anatomy

**Figure 5:**
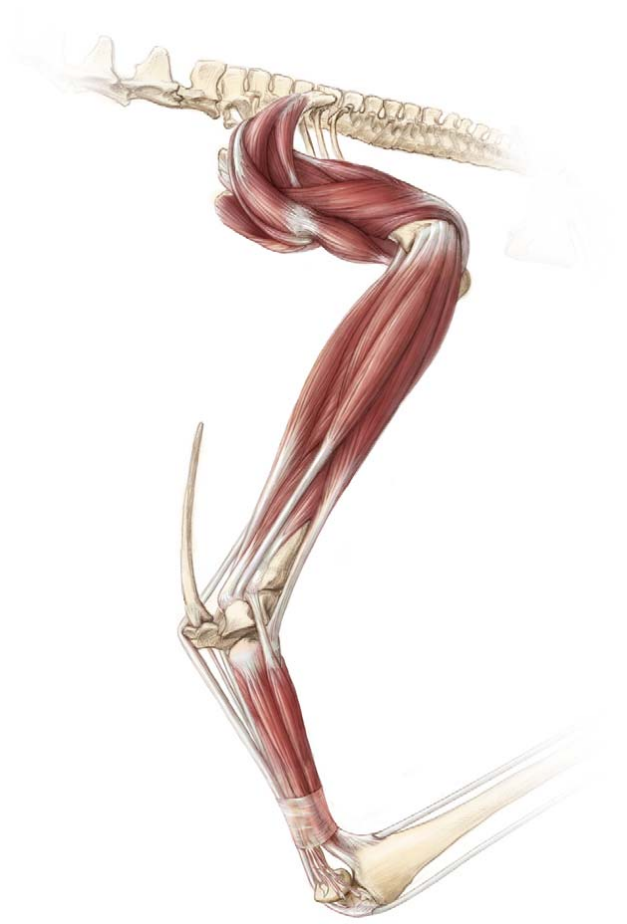
Lateral view of the forelimb musculature in *Anhanguera santanae*. Note that the forelimb musculature is extensive, and that the major muscle base used for flight is more distributed than that of birds. Unlike avian taxa, pterosaurs derived substantial flapping power from several groups of muscles around the chest and back (rather than the two primary muscles in birds), as well as the antebrachium and manus. Illustration by Julia Molnar, used with permission.

Along with being incredibly strong, the morphology of pterosaur bones also advocates a flighted lifestyle (Figs. 1, 3–4). Most obviously, all pterosaurs bear hypertrophied fourth manual digits that supported a thin but complex membrane in life [60]–[62], forming a very obvious wing. All pterosaurs bore robust, fused scapulacoracoids and, in derived, fully grown pterodactyloids, the anterior dorsal vertebrae fused into a rigid notarium, an adaptation to resisting bending and torsional forces on the body during aerial manoeuvres and flapping. Their sterna are deep and sculpted with a large cristospina projecting anteriorly, allowing for anchorage of large muscles involved in the flapping downstroke [73]. In many pterosaurs, azhdarchids especially, the coracoid flanges are also quite expansive, providing a wide origin *for m. coracobrachialis*, which also contributed to the flight stroke in pterosaurs. Their humeri are robust, bearing blocky extremities suited to resisting high stresses during takeoff and flight [41] and possess large, flange-like deltopectoral crests. Reconstructing the forelimb musculature (Fig. 5) indicates a robust set of proximal muscle groups, including large wing abductors and adductors [73]. This observation disagrees with the relative mass fractions reported by Henderson [25] that suggest relatively small amounts of mass associated with the appendicular anatomy, but we suggest that this stems from using outdated reconstructions (from Wellnhofer [6]) to map body shapes and proportions. The expected flight muscle fractions of large pterosaurs would have greatly exceeded the flight muscle fractions measured for most birds, in part because the forelimbs and pectoral girdle represent such a disproportionate percentage of pterosaur mass (Table 3): Strang *et al.* [74] estimated that over 40% of the mass of the ornithocheirid *Anhanguera piscator* was appendicular bone, muscle, and skin, for example.

**Table 3:** Relative masses of different giant pterosaur skeletal components (derived from [24]).

			Proportional bone mass (%)				
Taxon	Wingspan (m)	Predicted skeletal mass (g)	Skull and mandible	Cervical vertebrae	Torso	Forelimbs	Hindlimbs
*Pteranodon*	5.43	2553.54	0.29	0.07	0.20	0.41	0.04
*Quetzalcoatlus*	9.64	18034.65	0.17	0.11	0.15	0.47	0.10

Many of these features developed convergently with modern flying vertebrates and their relation to flight was recognised very early on in pterosaur research [6], [75]. So far as can be seen from their often fragmentary fossils, flight characteristics are also found in the skeletons of the largest azhdarchids, with the forelimb skeletal spar and muscle attachment sites accordingly more robust in larger pterosaurs than their smaller counterparts. *Pteranodon*, the only giant pterosaur for which the entire skeleton is known, certainly bears all the anatomical hallmarks of flight seen in smaller pterosaurs [12] and (as discussed in greater depth below) stands out as one of the most flight-adapted of all pterosaurs. The wings of *Q. northropi* and *Hatzegopteryx* possess particularly large deltopectoral crests and robust extremities, with the former also known to bear the same the membrane-supporting wing-finger typical of all pterosaurs [28]–[30]. Henderson [23] considers *Quetzalcoatlus* to have “peculiarly short wings”, but we suspect the wings of *Q. northropi* only appear reduced relative to the over-estimated body length in his *Q. northropi* model. Granted, azhdarchids do have unusual proportions that may produce the appearance of shortened wings (particularly their elongate heads and necks; shortened wing fingers and hypertrophied wing metacarpal), but their wingspans are not especially shorter than would be expected for any other lophocratian pterosaur of their size.

It is possible, however, that giant pterosaurs represent old, flightless individuals of a species that were capable of flight when younger, their flight anatomy simply being retained from a previous stage in their life history. However, if the maximum flight sizes suggested by Sato *et al.* [17] and Chatterjee and Templin [19] are correct at 41kg (5.1 m span) or 70 kg (6.65 m with ‘heavy’ estimates), then the largest azhdarchids would have grown up to six times the mass and twice the wingspan of their terminal flight size. It seems unlikely that enormous azhdarchids would continue to develop their physiologically expensive flight apparatus, and coincidentally with a mechanically appropriate scaling regime, throughout such extensive growth under flightless conditions. The same point is true, though to a lesser extent, for large *Pteranodon* that also bear obvious flight characteristics despite having exceeded the suggested wingspan limits of flight. If anything, the scaling regimes of pterosaur wings dictate that the flight characteristics of giant pterosaurs (the size of their deltopectoral crests, robustness of their joints) - become more exaggerated with size and age (e.g. [76]), precisely the opposite of what would be expected in animals that lost their flight ability as they grew older.

On a similar note, the suggested size-gap between giant pterosaurs and their smaller relatives, said to parallel that seen between flying birds and the flightless ratites, by Henderson [25] does not exist. Padian and Smith [77]; Buffetaut *et al.* [78]; Company *et al.* [79] and Ibrahim *et al.* [80] report azhdarchid material that represents individuals with spans between 6 and 9 m, neatly filling the gap between giant azhdarchids and better known, smaller individuals (e.g. [32]–[33]). Even if such a size gap was known, we are uncertain of its significance: a number of flightless birds are of comparable sizes to flighted species (e.g. kiwis, numerous gruiforms, most penguins), suggesting large size does not characterise flightlessness at all and, in any case, the existence of a ratite-to-flighted bird size gap is also questionable when extinct taxa are considered (e.g. dwarf emus). We conclude, therefore, that even the largest pterosaurs possess the same hallmarks of flight as smaller pterosaurs (as noted for *Hatzegopteryx* by Buffetaut *et al.* [30]) and, on grounds of comparative anatomy, they should be considered flighted.

#### Flight performance

Pterosaur bone mechanics and anatomy indicate they were at least capable of flight, but they do not necessarily advocate efficient, sustained flight: giant pterosaurs could exhibit the same limited flight abilities seen in ‘poor’ modern fliers (heavyset animals with high wing loading for which flight is an energetically costly, typically short-lived affair). Comparisons of wing ecomorphology of giant pterosaurs with modern vertebrate fliers (see discussion below; also Hazelhurst and Rayner [11] and Witton [24] for details) suggest they do not plot among such animals, however. Although their position on ecomorphospace plots vary with the mass estimations and wing areas used, the flight models of Brower and Veinus [81], Hazlehurst and Rayner [11], Chatterjee and Templin [19] and Witton [24] show giant pterosaurs to fall amongst competent fliers such as aerial predators or dynamic and static soarers (Fig. 2). Moreover, our flight range estimates suggest large pterosaurs were likely able to fly considerable distances under anaerobic power after launch to find external sources of lift, owing to their relatively large muscle fractions. This would be aided by their ability to reach high velocities upon takeoff [41], which would limit the required acceleration during climbout. Therefore, while the largest pterosaurs appear to exceed the size limits for continuous flapping flight by a volant animal, there is no reason to suspect that they could not fly long distances Rather, it is reasonable to expect that so long as giant pterosaurs launched within 1 to 2 kilometres of an external source of lift, they could then stay aloft by transitioning to a soaring-dominated mode of travel after an initial burst of anaerobic power.

#### Competence at other forms of locomotion

Any suggestion that giant pterosaurs were flightless needs to consider other means through which they could travel. Pterosaur terrestrial locomotion has received a wealth of interest in the last few decades (e.g. [12], [18], [82]–[86]) and pterosaurs are now generally considered to be adept terrestrial animals as well as skilled fliers. The terrestrial capability of *Pteranodon* and other ornithocheiroids remains controversial, however: a full review of the literature on this topic is beyond the scope of this work, but ornithocheiroids have been proposed to drag themselves along the ground on their bellies [87] or walk bipedally [12], [84] or quadrupedally with sprawled [20], [88] or parasagittally-held limbs [19], [89]. A key element in this controversy is the pronounced dichotomy in ornithocheiroid hindlimb and forelimb lengths: this produces a long, flight-efficient wing but gives dramatically different stride lengths in the fore and aft limbs when walking or running. A potential solution to this issue, bipedal walking [12], [84], has been criticised with suggestions that the hindlimbs are comparatively diminutive compared to the rest of the body, that the hindlimb musculature would achieve poor mechanical advantage if the pterosaur body was elevated to an erect bipedal pose [90], that the anteriorly-positioned centre of gravity (induced by the large forelimbs, flight muscles and skull [20], [88]) would render the animal unstable and that the wings could not be folded away neatly [88]. As such, it seems unlikely that any ornithocheiroid could sustain a bipedal stance for a great length of time and would have had to overcome the hindlimb-forelimb length dichotomy inherent in their quadrupedal gait for sustained terrestrial locomotion.

The semi-erect, sprawled-limbed model of ornithocheiroid quadrupedalism requires considerable medial rotation of the propodial forelimb bones or significant depression of the metacarpals at the carpus. As these actions contradict primarily uniaxial arthrological ranges predicted in ornithiocheiroids and other pterosaur forelimbs [12], [20], this posture is considered unlikely here. We see no reason to assume that ornithiocheiroids held their limbs any differently from the parasagittal posture indicated in trackways for other pterodactyloids ([83], [86], also Hyder *et al.* in prep.), but moving with such a pronounced difference in fore- and hindlimb length almost certainly hampered their terrestrial agility and speed. We suspect that ornithiocheiroids may have relied on different gaits more than pterodactyloids with relatively equate and adaptable limb lengths, particularly when attempting to move quickly. Employment of bipedal running, for instance, would permit faster, more efficient movement over land (and, we note, not be problematic in the manner that bipedal standing or walking is), as would saltatorial or bounding gaits, as-yet uninvestigated mechanisms of pterosaur locomotion. Full discussion of these ideas is beyond the scope of this work, but we note that pterosaurs – and especially ornithiocheiroids - bear the dichotomous limb lengths, short trunks, uniaxial limb motion and distally elongated antebrachial elements consistent with saltatorial habits [91], though they would differ dramatically from living saltators by using their forelimbs as the main propulsors. Pterosaurs do lack the large appendages and heavy tails of saltatorial animals, however, suggesting that if they did saltate, their speed or agility would be less than those of specialised saltators. In any case, both pterosaur limb sets are adapted for powerful leaping [18], [41], [70] and at least bounding gaits were probably attainable in all pterosaurs. Even with this choice of locomotory mechanisms, however, the limb length dichotomy of ornithocheiroids almost certainly places them among the least terrestrially-adept of all pterosaurs. This does not exclude the possibility that *Pteranodon* and other large ornithocheiroids were flightless as some birds are flightless but not particularly proficient terrestrial locomotors. These birds are typically secondarily adapted for alternative lifestyles however (e.g. swimming and diving), and *Pteranodon* shows no evidence for secondary adaptations of this kind. Not only does its pedal claw morphology indicate that more time was spent standing than floating [88], but *Pteranodon* lacks suitable appendicular anatomy for aquatic propulsion and probably was, at best, a limited swimmer. Indeed, the inhibitions on the non-volant abilities of *Pteranodon* probably stem from strong selection pressures on development of soaring-efficient flight apparatus.

Azhdarchids, by contrast, were recently suggested to have a greater terrestrial competence than any other pterosaur group [14], giving them much greater potential for a flightless existence than giant ornithocheiroids. Azhdarchid trackways indicate that their feet were short (a mechanically advantageous trait for walking animals), possessed soft-tissue pads around the metatarsal heads and heels and that their limbs were held directly under the body when walking [14], [93]. Their skeletons reveal atypically long femora and wing metacarpals that serve to lengthen the limbs for increased stride efficiency, while their pedal bones are unusually robust (Fig. 4; [12], [32], [26]). Taken together, these features indicate that azhdarchids were well adapted for a terrestrial locomotion and it seems likely that they spent much of their time grounded, particularly when foraging [14]. This suggests that a flightless existence was viable, though simply being a competent terrestrial animal does not exclude volant abilities.

#### Depositional settings of giant pterosaurs

The sedimentary contexts of both *Pteranodon* and azhdarchids have relevance in discussion of giant pterosaur flightlessness. Excellently-preserved *Pteranodon* fossils are known in considerable abundance hundreds of kilometres from the nearest contemporary palaeoshoreline [12]: it is highly unlikely that *Pteranodon* swam to such a location because, as discussed above, it bears no characteristics indicative of habitual swimming behaviour. Furthermore, the well-preserved, articulated nature of many specimens suggests they were not transported far after death. Terrestrial animals such as dinosaurs are found in the same deposits as *Pteranodon* but are much rarer and less completely preserved, indicating lengthy transportation prior to their deposition [94]. This points to live *Pteranodon* spending a lot of time around open-water, and its flight-compliant anatomy indicates that most of this time was spent flying above it. By contrast, most azhdarchids are found in terrestrially-derived sedimentary settings [14], [95], a finding that may be predicted if giant azhdarchids were flightless. While consistent with the flightless hypothesis, however, the terrestrial-skew of the azhdarchid fossil record can only serve as circumstantial evidence of azhdarchid flightlessness: there is no reason why azhdarchids, like many modern fliers, cannot simply preferentially inhabit terrestrial environments. Thus, whereas the depositional context of *Pteranodon* is singly telling about its habits, that of azhdarchids is of little use in determining their flight ability.

#### Summary: could giant pterosaurs fly?

There is virtually no indication from the anatomy, biomechanics, aerodynamic performance or depositional contexts of any giant pterosaurs that they had lost their ability to fly. This is particularly so for *Pteranodon*, an animal with anatomy so skewed towards a glide-efficient wing morphology that its terrestrial capabilities may have been lessened. The case is not so clear-cut for azhdarchids: as pterosaurs living within continental settings and apparently possessing good terrestrial abilities, they meet some criteria that may be expected of a flightless pterosaur. However, like *Pteranodon*, giant azhdarchids also possess skeletons that function well as flying apparatus and were almost certainly flighted as well.

These observations do not preclude the existence of flightless pterosaurs, however: it is entirely conceivable that some forms may have abandoned flight given the right environments and selection pressures. In our view, however, the pterosaur lineage closest to abandoning flight may not be giant at all but, rather, the considerably smaller basal pterosaur clade Dimorphodontidae (wingspans of 0.6–1.3 m [6]). *Dimorphodon* has been found to be a particularly heavyset pterosaur with relatively high wing loading, attributes found in modern fliers like rails and galliforms [24], [81] that find flight particularly energetically expensive. Given that *Dimorphodon* also possesses an unusually robust skeleton – including long limbs and well-developed appendages - it was probably also a competent terrestrial (or, more likely, scansorial – see Unwin [82]) animal that spent much of its time grounded (Hyder *et al.*, in prep). Dimorphodontids therefore possessed characteristics quite conducive to developing flightless habits and there seems little reason to assume that more derived members of this group could not have abandoned flight in the right conditions. We stress, however, that there is currently no evidence that any pterosaurs fully surrendered their flight abilities and, conversely, a wealth of evidence suggesting that all pterosaurs were flighted. Accordingly, this calls into question why some pterosaur flight models have predicted flightlessness in giant pterosaurs [17], [19], and we suspect that such errors are results represent *a priori* assumptions over the mechanical similarities of birds and pterosaurs.

### Are birds suitable analogues of giant pterosaurs?

Given that some attributes of bird and bat flight are directly comparable [63]–[64] it is not unreasonable to assume that some aspects of bird and pterosaur flight should not also be similar [11]. Whether all attributes of birds such as their mass, flapping frequencies and launch strategies can be directly applied to pterosaurs is questionable, however, and the use of purely avian-sourced data in calculations of pterosaur flight are probably responsible for some deductions that giant forms could not fly.

#### Launch mechanisms

Chatterjee and Templin [19], Sato *et al.* [17] and Henderson [25] assume that a critical aspect of flight, takeoff, was achieved with similar methods in pterosaurs and birds. The former authors assume a bipedal running start but, whereas Sato *et al.* assume that the wings were flapped vigorously whilst running, Chatterjee and Templin exclude this possibility on grounds that the wing membranes were attached to the hindlimbs and flapping could only begin after a leap into the air at the end of the run. Chatterjee and Templin state that an 85 kg *Q. northropi* could not take off with a run even using a strong headwind and full anaerobic power, suggesting to them that the masses of giant azhdarchids could not exceed 70 kg. Even at these low masses, however, giant azhdarchids ‘needed as much wind as they could get’ (p. 52) and would use downhill slopes to assist their launches. In contrast, Sato *et al.* place restrictions on the takeoff abilities of giant pterosaurs through their inference that flapping frequency decreases as mass increases. The procellariiforms of their study were found to flap at two frequencies, the lower used to sustain flight and the higher employed only when additional lift was needed during takeoff. Their regression lines of high- and low-frequency flapping against mass intersect at 41 kg (equating to a 5.1 m wingspan in their mass/wingspan regression), indicating that any soaring animal above this size would not generate enough thrust and lift to takeoff or maintain soaring flight. Henderson [25] suggests that large bustards (masses up to 22 kg) may simply represent the upper limit of flight given their difficulty with becoming airborne.

Of these three cases, Henderson's assertions in particular make several unsupported assumptions and conflict with the known modern and fossil diversity of flying birds. There have been no published accounts demonstrating that the largest living flying birds are at any kind of general mechanical limit for flight, and an “apparent difficulty in taking off” is both qualitative and anecdotal. Launch ability and rate is morphology specific - while bustards take short runs to launch [96], albatrosses of similar mass take much longer running starts [97] and turkeys of similar mass do not run at all to launch [67]. As a result, the largest extant flying birds cannot be taken to represent a flight limit for even other birds with slightly differing morphology. It is also worth noting that fossil birds of much greater size than living bustards, such as *Argentavis*, appear to have been capable of launch and flight [98]. We therefore emphasize that the limits of launch and flapping flight are contingent upon morphology; extrapolating limits from qualitative assessments of launch performance is, in our assessment, unwarranted and unsupported, especially when such extrapolations are made from birds to distantly related groups (such as pterosaurs).

**Figure 6:**
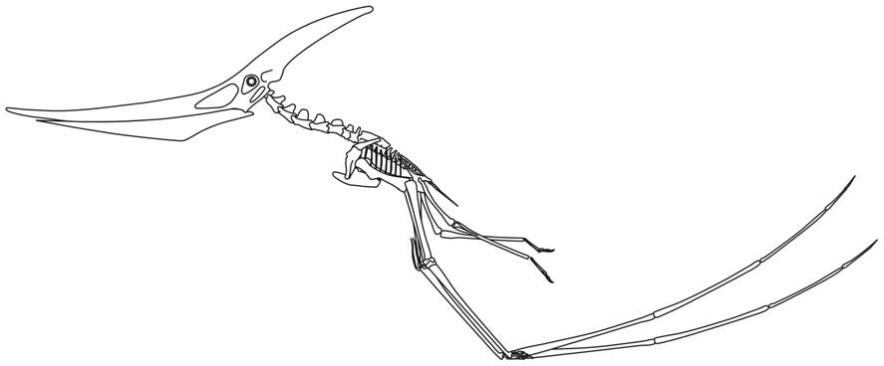
Skeletal reconstruction of a quadrupedally launching *Pteranodon*. Skeletal proportions based on Bennett [12]; kinematics from Habib [40].

The findings of Chatterjee and Templin [19] and Sato *et al.* [17] by contrast, provide interesting insight into the launch mechanics of hypothetical giant birds, but they may have little relevance to pterosaurs as the high-frequency flapping of bird takeoff is incomparable with probable methods of pterosaur launch. There is good evidence that pterosaurs launched from a standing, quadrupedal start in a superficially vampire bat-like fashion, vaulting over their forelimbs and using powerful flapping to gain altitude (Fig. 6, [41]). This launch strategy is entirely in keeping with the allometry of pterosaur limbs discussed above and explains why pterosaur femora are relatively slender at larger sizes compared to those of birds. Unlike birds, pterosaur femora are only partially responsible for generating power for flight and can, therefore, scale with lower exponents than their humeri (see Habib [41] for greater discussion of these points). The scaling allometry of the wing metacarpal is further evidence of this launch strategy: larger pterosaurs have disproportionately long wing metacarpals, a trait echoed in pterosaur ontogeny [76] as well as phylogeny. During quadrupedal launching, the increased length of these elements would increase the mechanical advantage of the vaulting pterosaur to assist takeoff, possibly of particular importance to relatively large, heavy pterosaurs. If pterosaurs did take off in such a fashion, applying entirely different avian takeoff strategies to pterosaurs reveals nothing about pterosaur flight. The possibility of quadrupedal launch in pterosaurs is particularly relevant here as it may have facilitated pterosaurs to become much larger than any avian fliers: using the more powerful and robust forelimbs for takeoff sets higher mass limits on launch capability [41] and will facilitate the evolution of much larger flying animals. In contrast to the low mass figures needed to launch *Q. northropi* using a bipedal method, a quadrupedally launching, 200–250 kg, 10 m span azhdarchid could easily launch from a standing start without use of downward slopes or headwinds (Habib, unpublished data). Thus, when modelled with non-avian launch kinematics, giant pterosaurs appear to have been strong, powerful launchers.

Even if this evidence is ignored, the cosmopolitan occurrence of azhdarchids in numerous terrestrial sedimentary basins [14], [99] counters arguments that specific environments or climates were essential for their flight. While gusty conditions may have been somewhat more consistent for the pelagic *Pteranodon*, wind strength varies considerably in terrestrial settings and it is unlikely that azhdarchids would be so abundant and successful if they required such consistent and specific weather conditions to fly. Likewise, there is no indication that azhdarchids were restricted to highland areas where launch-assisting slopes were common. Rather, the preferred terrestrial habits and flight-adapted skeletons of azhdarchids combine to suggest that even the largest azhdarchids could fly entirely under their own power regardless of local weather and landscape conditions. We concede that our azhdarchid flight model does suggest that flights of long-duration may be reliant on external sources of lift, but these occur through a variety of mechanisms in varied environments and climates [19]: we do not therefore see this as a limiting factor on azhdarchid flight.

#### Body proportions and masses

The mechanical incomparability of pterosaurs and birds is not limited to launch alone. Because bird flight mechanics differ vary with size and mass, phylogeny and ecology, selecting a group to model pterosaurs on is problematic and biases flight calculations. Averaging out attributes of bird flight may ignore important factors that may contribute to flight efficiency in one particular group, while extrapolating values from selected taxa imparts the particular constraints of their morphology into the flight analyses. As such, the constant comparisons of seabird flight mechanics to those of pterosaurs is of suspect validity. The taxa studied by Sato *et al.* [17] for instance (the streaked shearwater *Calonectris leucomelas*, white-chinned petrel *Procellaria aequinoctialis*, sooty albatross *Phoebetria fusca*, black-browed albatross *Thalassarche melanophrys*, wandering albatross *Diomedea exulans*), have highly-derived anatomy with very high aspect ratios, moderate wing loading (Table 1; [64], [100]–[101]) and pectoral and wing anatomy modified for energetically inexpensive gliding [102]–[103]. Their flight regularly employs head- or tailwinds to minimise flight costs [104] and these attributes combine to make procellariiforms proficient and highly-specialised dynamic soarers. In fact, procellariiform flight dynamics are even unique among living marine birds with similar planforms and body profiles; the gust-acceleration dynamic soaring method utilized by procellariiforms requires unique sensory adaptations in addition to specific morphological traits [65]. This, of course, is only one portion of the mosaic of bird ecomorphospace and the flight styles of every bird species are uniquely defined by mass, wing area, flight muscle masses, wing bone mechanics, wing loading, chord depth and numerous other factors [64], [105]. Accordingly, extrapolation of flight styles between avian species is relatively meaningless above the tightest taxonomic levels: recognising this, Sato *et al.* [17] state that their extrapolations apply to ‘phylogenetically similar species’ (p. 4), suggesting their conclusions can only apply to procellariiforms or, perhaps, animals that are highly convergent with procellariiforms.

**Figure 7:**
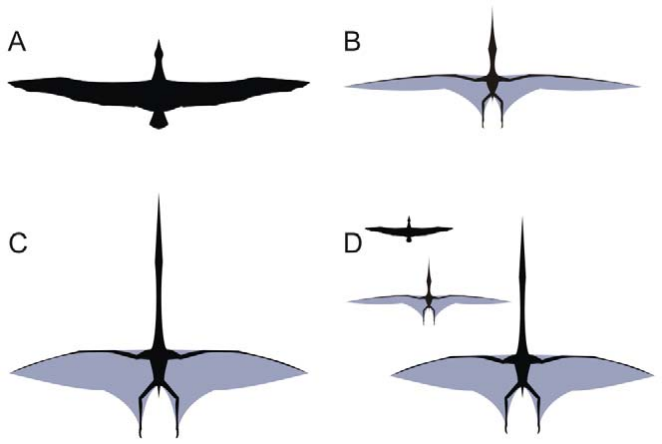
Soaring animal planforms compared. A, wandering albatross *Diomedea exulans*; B, the giant ornithocheiroid *Pteranodon*; the giant azhdarchid *Quetzalcoatlus*; D, shown to scale. See [24] for details of pterosaur wing planform reconstruction.

Although most pterosaurs have been proposed to be marine-bird analogues (e.g. 6), recent work suggests that seabird-like lifestyles were only one ecology exploited by pterosaurs and that they were probably considerably more diverse than previously appreciated [106]. Moreover, of the numerous pterosaur flight studies performed in the last 100 years, only one [24] has quantitatively demonstrated that some pterosaurs had procellariiform-like wing ecomophology (Fig. 2) and another found large pterosaurs to follow procellariiform-like glide patterns [19]: there is little other quantitative evidence that any pterosaurs were specifically procellariiform-like in life. Comparing the procellariiform body plan to that of pterosaurs may show why such data is scarce: procellariiform bodies are not particularly pterosaur-like (Figs. 4, 7) with longer, narrower wings that act independently of the hindlimbs, shorter necks, smaller heads and an entirely different pelvic and hindlimb morphology. The assumption by Sato *et al.* that ‘If those large pterosaurs had extremely slender bodies, more so than albatrosses and petrels, the maximum power of their muscles would have been less and their flapping capacity accordingly diminished’ (p. 4) is simply wrong: neither *Quetzalcoatlus* nor *Pteranodon* have bodies that are proportionally slenderer than those of procellariiforms, and nor were they under-muscled [21]–[24]. The oversize heads, necks and forelimbs of pterosaurs may give this impression, but these inflated elements represent exploitation of extensive pneumatisation in these features to attain the advantages of increased dimensions (e.g. greater wingspans, stride lengths, feeding envelopes, larger muscle attachment sites etc.). Accordingly, we should view these elements as particularly large, not the pterosaur body as being particularly small.

The differences between birds and pterosaurs become critical problems when extrapolating mechanical data from modern birds. Estimates of pterosaur mass from modern birds, for instance, are suspect for several reasons. The mass of *Pteranodon* estimated by Sato *et al.* [17] is much higher than previously published figures at 93 kg (‘heavy’ estimates for large *Pteranodon* range from 20–35 kg: see [24], table 1) whereas, by contrast, the 276 kg mass predicated for *Q. northropi* is close to mass estimations by Paul [22] and Witton [24]. A dataset of bird body and head lengths against mass were also used by Jersion [16] to estimate the mass of *Pteranodon* (20 kg) while Stein [107] used the wing area of another modern volant animal, a molossid bat, to estimate the mass of the same pterosaur at 15 kg. However, extrapolating the masses of modern animals to giant pterosaur-sizes does little else than predict the masses of equivalently-sized birds or bats, not pterosaurs themselves. Pterosaur body proportions and soft-tissue anatomy are very different from any modern volant animals: they lack feathers, for instance, that may account for over 10 per cent of avian body mass [69]. It seems unreasonable, therefore, to expect that the body forms of modern animals could be used to extrapolate pterosaur masses, and particularly so when the body forms in question is not especially pterosaur-like themselves.

We also note that extrapolating the mass of any modern flying animal (maximum span of 3 m) to giant pterosaur-sizes (spans of 7 or 10 m) requires data projection well beyond its upper range. Such extrapolation is extremely unreliable [108] and, in the case of the 93 kg Sato *et al. Pteranodon* estimate, may explain why these authors obtained a value that we consider to be almost certainly too high. Bramwell and Whitfield [87] estimated that a 7 m span *Pteranodon* would have a volume of around 40 L, giving the Sato *et al. Pteranodon* a body density of 2.235 g/cm^3^. As most birds have densities of 0.6–0.9 g/cm^3^ [108] and non-volant animals have densities of around 1 g/cm^3^ [91], this density is totally unrealistic unless, perhaps, *Pteranodon* was principally comprised of aluminium (2.7 g/cm^3^). For this reason, while the Sato *et al. Q. northropi* mass corresponds well with some recently published mass estimates for this animal, we do not consider the methodology behind the estimate to be reliable.

#### Flapping rates

Additional issues with proportional differences are found when specifics of flight are considered. Flapping frequency, the crux of the Sato *et al.* argument for giant pterosaur flightlessness, does not simply differ with mass or wingspan: modern birds demonstrate that span, body mass, and wing area, and relative muscle fractions can influence flapping rates considerably [58]. Thus, even among modern birds, applying universal limits of flapping frequency (and its subsequent influence on flight capacity or launch ability) is nearly impossible. In fact, flapping frequency scales to the 3/8 power of body mass if wing area and span are generated as separate scaling terms [58]. A migrating bird, for example, flaps more rapidly at the beginning of a migration than at the end (as its mass declines [66]). However, wing area and span correlate with body mass when compared across species [110], which means larger bird species do tend to flap more slowly than smaller taxa, but only when there is a high degree of geometric similarity between the comparison taxa (even then, the relationship is most applicable for continuous, steady state flapping). For example, while the large procellariiform taxa used in the Sato *et al.* [17] dataset are running launchers with low flapping frequencies, similarly sized burst flyers, such as wild turkeys, can launch vertically from a standstill, and flap rapidly [67]. This highlights an additional problem in deriving pterosaur performance from the scaling of flapping capacity in a specific group of birds: muscle physiology is variable among taxa and also scales with size [23]. It is very reasonable to think that large pterosaurs might have utilized relatively large fractions of high power fast oxidative or fast glycolytic muscle fibers (Cunningham, pers com) and, as such, the burst performance of large pterosaurs might have exceeded that seen in many bird species. Furthermore, there is no reason to presume that giant pterosaurs flapped continuously for long periods of time: our flap-gliding analysis suggests the flight muscle capacity of giant pterosaurs was utilized primarily for launch and climb out, with long-distance flight sustained mostly by external energy sources (i.e. unpowered flight sustained by soaring mechanisms, such as ridge shears and thermal columns).

Direct evidence that pterosaurs and seabirds had different flapping kinematics is provided with a comparison between the lengths of their functional wing regions (see Prondvai and Hone [111]). Pterosaur wings are constructed with different functional proportions than those of procellariiforms (Fig. 4D) with the brachial region (corresponding to the humeral length) relatively longer in procellariiforms, but the antebrachial region (ulna length in procellariiforms; unlna+syncarpus+wing metacarpal in pterosaurs) proportionally longer in pterosaurs. This proximal wing region dictates the size and area of the propatagium and, because of the importance of this element to generating lift [112] , its relative size and chord will affect flight performance considerably. The distal wing region (manus and primary feather length in procellariiforms; wing finger in pterosaurs) is of similar size in procellariiforms and azhdarchids but much longer in pteranodontians. The distal wing is of primary importance in generating propulsion during flight and, again, its variation across flying animals influences their flight performance and flapping mechanics [91]. As the length of these regions and the articulations between them dictate the shape of the wing during the flap cycle, the degree of span control and the shapes assumed by the wing during aerial manoeuvres, it seems unlikely that pterosaurs and bird wings of such differing proportions would generate comparable flapping mechanics.

There appears, therefore, to be many pitfalls in using birds as direct mechanical analogues of pterosaurs. The functional opportunities afforded by disparate morphologies need to be considered by workers attempting to investigate the flight mechanics of any particular animal group, including those attempting to establish the maximum sizes of flying animals. Along with the other issues outlined above, it demonstrates that seabirds cannot be reliably used to deduce details of pterosaur flight, nor, for that matter, the maximum size of any flying creature other than large, seabird-like forms.

### Is there a ‘generic’ giant pterosaur? *Quetzalcoatlus* and *Pteranodon* compared

Many authors appear to regard different pterosaur species as variations on the same basic bauplan, differing in wingspan and mass but otherwise locomoting and living in very similar ways (e.g. [5]–[6], [11], [17], [19], [112]–[113]). This notion is clearly flawed: as indicated above, there is no ‘generic’ pterosaur body plan or flight style in the same way that there is no ‘standard’ mammalian or avian bauplan or method of locomotion. That some studies (e.g. [17]) have only considered pterosaur wingspans and masses as variables when discussing their soaring ability ignores important factors that vary between species and conclusions based on such comparisons are likely to be oversimplified and correspondingly wrong.

Comparing the anatomy of giant pterosaurs demonstrates this well (Figs. 4, 6). Apart from large size, there are few morphological similarities between these taxa. The largest *Pteranodon* spanned around 7.25 m [12] with wing fingers that occupied 66 per cent of each wing. The glenoid is located dorsally on the scapulacoracoid, meaning that most of the body hung beneath the wings during flight (corresponding to the ‘upper-decker’ configuration of Frey *et al.* [114]). The neck, head and particularly the wings are large, rendering the body and hindlimbs proportionally small (the latter being 20 per cent of the wingspan). Because the wings are so long in comparison to the rest of the body, *Pteranodon* probably had a comparatively low mass for its wingspan [24]. *Quetzalcoatlus* is substantially larger than *Pteranodon* with a likely wingspan of 10–11 m ([28], also see above). The wings are proportionally shorter than those of *Pteranodon* with a wing finger that occupies 47 per cent of the wing length [34]. Combined with a relatively long fourth metacarpal, the relative contributions of the proximal and distal elements to the wing are quite different in these taxa (Fig. 4D). The *Quetzalcoatlus* glenoid is situated approximately mid-height on the body (‘middle-decker’ of Frey *et al.* [114]) and indicates that the relative dorsal musculature, and therefore relative upstroke power, was greatly expanded in *Quetzalcoatlus* relative to *Pteranodon*. For its wingspan, the *Quetzalcoatlus* hindlimbs and neck are relatively large (Tables 2 and 3; note that the largest neck vertebra in *Quetzalcoatlus* sp. is twice as strong as its femora!), meaning *Quetzalcoatlus* may have been relatively heavy for its wingspan.

The different morphology of these forms dictates that their flight performance must have also differed. Unfortunately, few studies have attempted to model the flight of both these giant pterosaurs, but it can be assumed that the proportional differences in wing-bone lengths (Fig. 4D) dictate that the wings of *Pteranodon* and *Quetzalcoatlus* would flex at different points during flap cycles and, in turn, affect the flapping kinematics and vortex generation of the two species for reasons discussed above. Several aspects of the morphology seen in *Quetzalcoatlus*, including its relatively expanded upstroke power (see below), expanded inboard wing proportions and high forelimb bone failure loads indicate that it was capable of producing higher transient lift coefficients than *Pteranodon*, and likely demonstrated proportionately better launch and landing performance. The longer body and legs of *Quetzalcoatlus* could create a deep, low-aspect wing that would generate greater lift during takeoff (assuming ankle-attached brachiopatagia – see Elgin and Hone [115]), while the smaller *Pteranodon* body and wings were narrower and produced less lift when launching but were more glide-efficient [100]. Alternative planforms for *Quetzalcoatlus*, in which the turn to the ankle is sharper and the outboard wing was narrower, still produce a bauplan better adapted to rapid bursts of flapping and tight manoeuvres than the planform seen in *Pteranodon*, despite *Quetzalcoatlus northropi* being nearly five-times more massive.

*Pteranodon* has received a wealth of aeronautical attention (e.g. [19], [87], [113], [116]), but few computations of *Quetzalcoatlus* flight have been published. The flight model of Chatterjee and Templin [19] suggests both *Pteranodon* and *Quetzalcoatlus* had flight comparable to that of albatrosses, but the inconsistent methodology, unrealistic mass estimations and questionable wing shapes (see [115]) used by these authors casts doubts on the validity of their results [24], [117]. Indeed, the *Quetzalcoatlus* and *Pteranodon* masses and wing areas proposed by Chatterjee and Templin [19] do not plot anywhere near the same ecomorphospace as albatrosses when factored into the wing loading and aspect ratio principle component analyses of Norberg and Rayner [63], Rayner [64] and Hazlehurst and Rayner [11] (Fig. 2). Rather, they plot in adaptive space not occupied by any modern vertebrate fliers, suggesting their flight styles are not represented in the modern day if the attributes calculated to them by Chatterjee and Templin are accurate. It is noteworthy, however, that the Chatterjee and Templin *Pteranodon* and *Quetzalcoatlus* occupy very different ecomorphospace on the PCA plot, as different from each other as, for instance, modern owls and gulls. The *Pteranodon* of Witton [24] was found to plot in the same flight adaptive zone as procellariiforms, modelling it as a glide-efficient dynamic soarer well-suited for a pelagic life in open-marine settings. The abundance and exclusivity of *Pteranodon* material in open-marine sediments [12] corroborates this interpretation. The wing loading of *Pteranodon* in this study was proportionally higher than that of *Quetzalcoatlus*, suggesting *Pteranodon* was also, relatively speaking, the faster flyer. *Quetzalcoatlus* plotted in the same ecomorphospace as condors and storks [24], supporting the suggestion that it was a static-soarer adapted for flight in terrestrial environments [29]. As with the depositional context of *Pteranodon* supporting a pelagic, ocean-going lifestyle, the taphonomic bias of azhdarchids towards continentally-derived sediments supports terrestrially-adapted flight [14]. In addition, these conclusions are supported by other functional studies of *Pteranodon* and *Quetzalcoatlus* [12], [14] and are consistent with their proposed lifestyles. Note, however, that when making such comparisons between pterosaurs and similarly loaded birds, tip slotting used by many inland birds during slow flight should increase the effective aspect ratio of the wing – as a result, a pterosaur with a slightly higher raw aspect ratio actually approaches the same performance as a bird with a lower raw AR, at least within the range of wing shapes where tip slots are utilized by avian species (at aspect ratios exceeding 12 tip slots are absent within living birds [118]. This is presumably the ratio at which the induced drag mediation is no longer favourable, relative to profile drag costs, even at relatively low speeds). This same observation has been made in passing by Cunningham and Pennycuick (pers comm.), but appears to be rarely indicated in the literature.

The uniting character of large size dictates that some aspects of all giant pterosaur flight would be shared, however. It is unlikely that any giant pterosaurs would need to flap continuously to remain aloft (as commented by Paul MacCready in 1984 while constructing his replica of *Quetzalcoatlus*) and, indeed, it is likely that the largest pterosaurs were incapable of flapping continuously for long periods. Their large size implies rapid cruising speeds and substantial anaerobic capacity [23] and, as discussed above, aspects of pterosaur skeletal morphology, especially within azhdarchids, also suggest an ability to flap powerfully for short bursts. Taking *Quetzalcoatlus* as an example, using the relatively broad planform suggested in Figure 7, a 200 kg mass and the flight equations from Pennycuick [58] the expected maximum range speed exceeds 30 m/s. A narrower planform and/or a heavier mass both produce even greater expected cruising speeds (maximum range speed rises with increased wing loading, as does best glide speed). At these speeds, with an anaerobic burst of only 30 to 60 seconds to power flapping, *Quetzalcoatlus* would have a nearly 2 kilometer radius in which to find an external source of lift. As such, while the largest pterosaurs would require reasonable soaring conditions somewhere in the vicinity of a given launch point in order to stay aloft for a long period of time, they would have a very wide range within which to locate and utilize such conditions.

Thus, anatomical differences and pterosaur flight studies indicate that giant pterosaurs flew in different fashions, and the application of one flight style to all giant pterosaurs is almost certainly incorrect. We note in addition that at least two studies have found pterosaur flight to be diverse, with pterosaurs representing, among others, marine-soarers, inland-soarers, generalist fliers, aerial-predators and burst fliers (Fig. 2; [11], [24]). Thus, like birds and bats, differences in pterosaur body form represent specific adaptations to flight and, in all likelihood, other aspects of pterosaur functional morphology, such as their terrestrial locomotion, were similarly varied. The concept of a ‘generic’ pterosaur bodyplan and universal locomotory style, giant or otherwise, should be entirely abandoned.

### Conclusions

While the conclusions on giant pterosaur flight by Chatterjee and Templin [19], Sato *et al.* [17], Henderson [25] and other workers using avian analogues for pterosaurs are not without merit, we find that they leave themselves open to criticism by not considering alternative data sources when making inferences about the palaeobiology of extinct animals. Giant pterosaur anatomy, functional morphology and, in the case of *Pteranodon* at least, sedimentary context all indicate that they were flighted animals and, likewise, clear anatomical distinctions between birds and pterosaurs indicate that only basic mechanical details can be treated interchangeably. It is also noteworthy that while the discussion here has focused exclusively on pterosaurs, several groups of extinct but clearly volant, soaring birds (teratorns, pelagornithids) also achieved sizes considerably larger than the suggested flight limitations critiqued here [98], [119]–[120]: many of the arguments made above apply equally to these birds and their existence provides additional refutation to the conclusions made about maximum flying animal size, and particularly those of Sato *et al.* [17] and Henderson [25].

In closing, we hope this study highlights two main issues, Firstly, while comparing birds with pterosaurs is probably more informative than the clinical engineering approaches taken to pterosaur flight research by many 20^th^ century workers, the pterosaur-bird analogy can be stretched too far. Pterosaur anatomy is completely unique, and any study of its function that ignores this individuality is likely to be flawed. Secondly, we stress that setting global limits for flying animals will always be fraught with intense difficulty and uncertainty because mechanical limits for any given morphology do not necessarily apply to other bauplans. In all likelihood, there is no universal maximum for any major characteristic, including size, that can be applied to all flying vertebrates, or even most of them.

## References

[1] Bennett SC (1996). The phylogenetic position of the Pterosauria within the Archosauromorpha.. Zoological Journal of the Linnaean Society.

[2] Benton MJ (1999). *Scleromochlus taylori* and the origin of dinosaurs and pterosaurs.. Philosophical Transactions of the Royal Society, London B.

[3] Hone DWE, Benton MJ (2008). Contrasting supertrees and total-evidence methods: pterosaur origins.. Zitteliana.

[4] Brown B (1943). Flying reptiles.. Natural History.

[5] Wellnhofer P (1978). Handbuch der Paläoherpetologie. Teil 19: Pterosauria..

[6] Wellnhofer P (1991). The Illustrated Encyclopaedia of Pterosaurs..

[7] Padian K (1985). The origins and aerodynamics of flight in extinct vertebrates.. Palaeontology.

[8] Unwin DM (2005). The Pterosaurs from Deep Time..

[9] Frey E, Martill DM (1996). A reappraisal of *Arambourgiania* (Pterosauria, pterodactyloidea): one of the world's largest flying animals.. Neuhes Jahrbuch für Geologie und Paläontologie, Abhandlugen.

[10] Martill DM, Frey E, Sadaqah RM, Khoury HN (1998). Discovery of the holotype of the giant pterosaur *Titanopteryx philadephia* Arambourg, 1959 and the status of *Arambourgiania* and *Quetzalcoatlus*.. Neues Jahrbuch für Geologie and Paläeontologie, Abhandlungen.

[11] Hazlehurst GA, Rayner JMV (1992). Flight characteristics of Triassic and Jurassic Pterosauria: an appraisal based on wing shape.. Paleobiology.

[12] Bennett SC (2001). The osteology and functional morphology of the Late Cretaceous pterosaur *Pteranodon*.. Palaeontographica Abteilung A.

[13] Witmer LM, Chatterjee S, Franzosa J, Rowe T (2003). Neuroanatomy of flying reptiles and implications for flight, posture and behaviour.. Nature.

[14] Witton MP, Naish D (2008). A reappraisal of azhdarchid pterosaur functional morphology and paleoecology.. PLoS ONE.

[15] Claessens LPAM, O'Connor PM, Unwin DM (2009). Respiratory Evolution Facilitated the Origin of Pterosaur Flight and Aerial Gigantism.. PLoS ONE.

[16] Jerison HJ (1973). Evolution of the brain and intelligence..

[17] Sato K, Sakamoto K, Watanuki Y, Takahashi A, Katsumata N (2009). Scaling of soaring seabirds and implications for flight abilities of giant pterosaurs.. PLoS ONE.

[18] Padian K (1983). Osteology and functional morphology of *Dimorphodon macronyx* (Buckland) (Pterosauria: Rhamphorhynchoidea) based on new material in the Yale Peabody Musuem.. Postilla.

[19] Chatterjee S, Templin RJ (2004). Posture, Locomotion and Palaeoecology of Pterosaurs.. Geological Society of America Special Publication.

[20] Wilkinson MT (2008). Three dimensional geometry of a pterosaur wing skeleton, and its implications for aerial and terrestrial locomotion.. Zoological Journal of Linnaean Society.

[21] Paul GS (1991). The many myths, some old, some new, of dinosaurology.. Modern Geology.

[22] Paul GS (2002). Dinosaurs of the Air: The Evolution and Loss of Flight in Dinosaurs and Birds..

[23] Marden JH (1994). From damselflies to pterosaurs: how burst and sustainable flight performance scale with size.. American Journal of Physiology.

[24] Witton MP (2008). A new approach to determining pterosaur body mass and its implications for pterosaur flight.. Zitteliana.

[25] Henderson DM (2010). Pterosaur body mass estimates from three-dimensional mathematical slicing.. Journal of Vertebrate Paleontology.

[26] Unwin DM, Buffetaut E, Mazin JM (2003). On the phylogeny and evolutionary history of pterosaurs.. Evolution and Palaeobiology of Pterosaurs, Geological Society Special Publication.

[27] Carpenter K, Unwin D, Cloward K, Miles C, Miles C, Buffetaut E, Mazin JM (2003). A new scapognathine from the upper Jurassic Morrison Formation of Wyoming, USA.. Evolution and Palaeobiology of Pterosaurs, Geological Society Special Publication.

[28] Langston W (1981). Pterosaurs.. Scientific American.

[29] Lawson DA (1975). Pterosaur from the Latest Cretaceous of West Texas: discovery of the largest flying creature.. Science.

[30] Buffetaut E, Grigorescu D, Csiki Z (2002). A new giant pterosaur with a robust skull from the latest Cretaceous of Romania.. Naturwissenschaften.

[31] Buffetaut E, Grigorescu D, Csiki Z, Buffetaut E, Mazin JM (2003). Giant azhdarchid pterosaurs from the terminal Cretaceous of Transylvania (western Romania).. Evolution and Palaeobiology of Pterosaurs, Geological Society Special Publication.

[32] Cai Z, Wei F (1994). *Zhejiangopterus linhaiensis* (Pterosauria) from the Upper Cretaceous of Linhai, Zhejiang, China.. Vertebrata PalAsiatica.

[33] Kellner AWA, Langston W (1996). Cranial remains of *Quetzalcoatlus* (Pterosauria, Azhdarchidae) from Late Cretaceous sediments of Big Bend National Park.. Journal of Vertebrate Paleontology.

[34] Unwin DM, Lü J, Bakhurina NN (2000). On the systematic and stratigraphic significance of pterosaurs from the Lower Cretaceous Yixian Formation (Jehol Group) of Liaoning, China.. Mitteilungen aus dem Museum für Naturkunde Berlins, Geowissenschaftliche Reihe.

[35] Steel L, Martill DM, Kirk JRJ, Anders A, Loveridge RF, Frey E, Martin JG (1997). *Arambourgiania philidelphiae*: giant wings in small halls.. The Geological Curator.

[36] Wellnhofer P (1970). Die Pterodactyloidea (Pterosauria) der Oberjura-Plattenkalke Süddeutschlands.. Bayerische Akademie der Wissenschaften, Mathematisch- Wissenschaftlichen Klasse, Abhandlugen.

[37] Parrish JM, Carrano MT, Gaudin TJ, Blob RW, Wible JR (2006). The origins of high browsing and the effects of phylogeny and scaling on the neck length in sauropodomorphs.. Amniote paleobiology: perspectives on the evolution of mammals, birds and reptiles.

[38] Tschanz K (1988). Allometry and heterochrony in the growth of the neck of Triassic prolacertiform reptiles.. Palaeontology.

[39] O'Keefe FR, Hiller N (2006). Morphologic and ontogenetic patterns in elasmosaur neck length, with comments on the taxonomic utility of neck length variables.. Paludicola.

[40] Pereda Suberbiola X, Bardet N, Jouve S, Iarochéne M, Bouya B, Buffetaut E, Mazin JM (2003). A new azhdarchid pterosaur from the Late Cretaceous phosphates of Morocco.. Evolution and Palaeobiology of Pterosaurs, Geological Society Special Publication.

[41] Habib MB (2008). Comparative evidence for quadrupedal launch in pterosaurs.. Zitteliana.

[42] Carter DR (1978). Anisotropic analysis of strain rosette information from cortical bone.. Journal of Biomechanics.

[43] Rubin CT, Lanyon LE (1982). Limb mechanics as a function of speed and gait: A study of functional strains in the radius and tibia of horse and dog.. Journal of Experimental Biology.

[44] Swartz SM, Bennett MB, Carrier DR (1992). Wing bone stresses in free flying bats and the evolution of skeletal design for flight.. Nature.

[45] Biewener AA, Dial KP (1995). In vivo strain in the humerus of pigeons (*Columba livia*) during flight.. Journal of Morphology.

[46] Blob RW, Biewener AA (1999). In vivo locomotor strain in the hindlimb bones of *Alligator mississippiensis* and *Iguana iguana*: implications for the evolution of limb bone safety factor and non-sprawling limb posture.. Journal of Experimental Biology.

[47] Carrano MT, Biewener AA (1999). Experimental alteration of limb posture in the chicken (*Gallus gallus*) and its bearing on the use of birds as analogs for dinosaur locomotion.. Journal of Morphology.

[48] Gere JM, Timoshenko SP (1990). Mechanics of Materials.

[49] Selker F, Carter DR (1989). Scaling of long bone fracture strength with animal mass.. Journal of Biomechanics.

[50] Polk JD, Demes B, Jungers WL, Biknevicius AR, Heinrich RE, Runestad JA (2000). A comparison of primate, carnivoran and rodent limb bone cross-sectional properties: are primates really unique?. Journal of Human Evolution.

[51] Ruff CB (2000). Body size, body shape, and long bone strength in modern humans.. Journal of Human Evolution.

[52] Ruff CB (2002). Long bone articular and diaphyseal structure in Old World monkeys and apes, I: Locomotor effects.. American Journal of Physical Anthropology.

[53] Habib M, Ruff CB (2008). The effects of locomotion on the structural characteristics of avian limb bones.. Zoo J Linn Soc.

[54] Kirkpatrick SJ (1994). Scale effects on the stresses and safety factors in the wing bones of birds and bats.. Journal of Experimental Biology.

[55] Swartz SM, Middleton KM (2008). Biomechanics of the bat limb skeleton: Scaling, material properties, and mechanics.. Cells, Tissues and Organs.

[56] Biewener AA (1982). Bone strength in small mammals and bipedal birds: do safety factors change with body size?. Journal of Experimental Biology.

[57] McGowen MR, Padian K, de Sosa MA, Harmon RW (2002). Description of *Montanazhdarcho minor*, an azhdarchid pterosaur from the Two Medicine Formation (Campanian) of Montana.. Paleobios.

[58] Pennycuick CJ (2008). Modelling the Flying Bird.

[59] Song A, Xiadong T, Israeli E, Galvao R, Bishop K, Swartz S, Breuer K (2008). The aero-mechanics of low aspect ratio compliant membrane wings, with applications to animal flight..

[60] Padian K, Rayner JMV (1993). The wings of pterosaurs.. American Journal of Science.

[61] Frey E, Tischlinger H, Buchy MC, Martill DM, Buffetaut E, Mazin JM (2003). New specimens of Pterosauria (Reptilia) with soft parts with implications for pterosaurian anatomy and locomotion.. Geological Society Special Publication.

[62] Kellner AWA, Wang X, Tischlinger H, Campos DAC, Hone DWE (2009). The soft tissue of *Jeholopterus* (Pterosauria, Anurognathidae, Batrachognathinae) and the structure of the pterosaur wing membrane.. Proceedings of the Royal Society B.

[63] Norberg UML, Rayner J (1987). Ecological morphology and flight in bats (Mammalia; Chiroptera): wing adaptations, flight performance, foraging strategy and echolocation.. Philosophical Transactions of the Royal Society, London B.

[64] Rayner JM (1988). Form and function in avian flight.. Current Ornithology.

[65] Pennycuick C (2002). Gust soaring as a basis for the flight of petrels and albatrosses (Procellariiformes).. Avian Science.

[66] Pennycuick CJ (1979). Energy costs of locomotion and the concept of “foraging radius”. Serengeti: dynamics of an ecosystem.. University of Chicago Press.

[67] Askew GN, Marsh RL (2002). Muscle designed for maximum short-term power output: quail flight muscle.. The Journal of Experimental Biology.

[68] Butler RJ, Barratt PM, Gower DJ (2009). Postcranial skeletal pneumaticitiy and air-sacs in the earliest pterosaurs.. Biology Letters.

[69] Prange HD, Anderson JF, Rahn H (1979). Scaling of skeletal mass to body mass in birds and mammals.. The American Naturalist.

[70] Bennett CS (1997). The arboreal leaping theory of the origin of pterosaur flight.. Historical Biology.

[71] McMahon TA (1975). Allometry and biomechanics: limb bones in adult ungulates.. American Naturalist.

[72] Christensen P (2002). Mass allometry of the appendicular skeleton in terrestrial mammals.. Journal of Morphology.

[73] Bennett SC (2003). Morphological evolution of the pectoral girdle in pterosaurs: myology and function. In: Buffetaut E, Mazin JM, editors. Evolution and Palaeobiology of Pterosaurs.. Geological Society Special Publication.

[74] Strang KA, Kroo I, Gerritsen M, Delp S (2009). Efficient flight of pterosaurs – an unsteady aerodynamic approach..

[75] Taquet P, Padian K (2004). The earliest known restoration of a pterosaur and the philosophical origins of Cuvier's *Ossemens Fossiles*.. Comptes Rendus.Palaevol.

[76] Codorinú L, Chiappe LM (2004). Early juvenile pterosaurs (Pterodactyloidea: *Pterodaustro guinazui*) from the Lower Cretaceous of central Argentina.. Canadian Journal of Earth Sciences.

[77] Padian K, Smith M (1992). New light on Late Cretaceous pterosaur material from Montana.. Journal of Vertebrate Paleontology.

[78] Buffetaut E, Laurent Y, Le Lœuff J, Bilotte M (1997). A terminal Cretaceous giant pterosaur from the French Pyrenees.. Geological Magazine.

[79] Company J, Unwin DM, Pereda Suberbiola X, Ruiz-Omeñaca JI (2001). A giant azhdarchid pterosaur from the latest Cretaceous of Valencia, Spain – the largest flying creature ever?. Journal of Vertebrate Paleontology.

[80] Ibrahim N, Unwin DM, Martill DM, Baidder L, Zouhri S (2010). A new pterosaur (Pterodactyloidea, Azhdarchidae) from the Upper Cretaceous of Morocco.. PLoS ONE.

[81] Brower JC, Venius J (1981). Allometry in pterosaurs.. The University of Kansas Paleontological Contributions.

[82] Unwin DM (1988). New remains of the pterosaur *Dimorphodon* (Pterosauria: Rhamphorhynchoidea) and the terrestrial ability of early pterosaurs.. Modern Geology.

[83] Unwin DM (1997). Pterosaur tracks and the terrestrial ability of pterosaurs.. Lethaia.

[84] Bennett SC (1990). A pterodactyloid pterosaur from the Santana Formation of Brazil: implications for terrestrial locomotion.. Journal of Vertebrate Paleontology.

[85] Lockley MG, Logue TJ, Moratalla JJ, Hunt APP, Schultz J, Robinson JM (1995). The fossil trackway *Pteraichnus* is pterosaurian, not crocodilian: implications for the global distribution of pterosaur tracks.. Ichnos.

[86] Mazin JM, Billon-Bruyat J, Hantzepergue P, Lafaurie G, Buffetaut E, Mazin JM (2003). Ichnological evidence for quadrupedal locomotion in pterodactyloid pterosaurs: trackways from the late Jurassic of Crayssac.. Evolution and Palaeobiology of Pterosaurs, Geological Society Special Publication.

[87] Bramwell CD, Whitfield GR (1974). Biomechanics of *Pteranodon*.. Philosophical Transactions of the Royal Society of London.

[88] Wellnhofer P (1988). Terrestrial locomotion in pterosaurs.. Historical biology.

[89] Henderson D, Unwin DM (1999). Mathematical and computational model of a walking pterosaur.. Journal of Vertebrate Paleontology.

[90] Fastnacht M (2005). The first dsungaripterid pterosaur from the Kimmeridgian of Germany and the biomechanics of pterosaur long bones.. Acta Palaeontologica Polonica.

[91] Hildebrand M (1995). Analysis of vertebrate structure, Fourth Edition..

[92] Smith AC (2007). Pteranodont claw morphology and its implications for aquatic locomotion..

[93] Hwang KG, Huh M, Lockley MG, Unwin DM, Wright JL (2002). New pterosaur tracks (Pteraichnidae) from the Late Cretaceous Uhangri Formation, S. W. Korea.. Geological Magazine.

[94] Everhart MJ (2005). Oceans of Kansas: A Natural History of the Western Interior Sea..

[95] Buffetaut E, Clarke JB, Le Lœuff J (1996). A terminal Cretaceous pterosaur from the Corbiéres (southern France) and the problem of pterosaur extinction.. Bulletin de la Societe Geologique de France.

[96] Prozesky OPM (1970). *A field guide to the birds of southern Africa*..

[97] Tickell WLN (2000). *Albatrosses*..

[98] Chatterjee S, Templin RJ, Campbell KE (2007). The aerodynamics of *Argentavis*, the world's largest flying bird from the Miocene of Argentina.. Proceedings of the National Academy of Science.

[99] Averianov A (2007). New records of azhdarchids (Pterosauria, Azhdarchidae) from the Late Cretaceous of Russia, Kazakhstan, and Central Asia.. Paleontological Journal.

[100] Pennycuick CJ (1971). Gliding flight of the white-backed vulture *Gyps africanus*.. Journal of Experimental Biology.

[101] Pennycuick CJ (1987). Flight of auks (Alcidae) and other northern seabirds compared with southern procellariiformes: ornithodolite observations.. Journal of Experimental Biology.

[102] Hui CA (2002). Avian furcula morphology may indicate relationships of flight requirements among birds.. Journal of Morphology.

[103] Meyers RA, Stakebake EF (2005). Anatomy and histochemistry of spread-wing posture in birds. 3. Immunohistochemistry of flight muscles and the “shoulder lock” in albatrosses.. Journal of Morphology.

[104] Spear LB, Ainley DG (1997). Flight behaviour of seabirds in relation to wind direction and wing morphology.. Ibis.

[105] Templin RJ (2000). The spectrum of animal flight: from insects to pterosaurs.. Progress in Aerospace Sciences.

[106] Witton MP (2008). The Palaeoecology and Diversity of Pterosaurs..

[107] Stein RS (1975). Dynamic analysis of *Pteranodon ingens*: a reptilian adaptation to flight.. Journal of Paleontology.

[108] Schmidt-Nielsen K (1984). Scaling: why is animal size so important?.

[109] Seamans TW, Hamershock DW, Bernhardt GE (1995). Determination of body density for twelve bird species.. Ibis.

[110] Greenewalt CH (1975). The Flight of Birds: The Significant Dimensions, Their Departure from the Requirements for Dimensional Similarity, and the Effect on Flight Aerodynamics of That Departure.. Transactions of the American Philosophical Society.

[111] Prondvai E, Hone DWE (2009). New models for the wing extension in pterosaurs.. Historical Biology.

[112] Brown RE, Cogley AC (1996). Contributions of the propatagium to avian flight.. The Journal of Experimental Zoology.

[113] Hankin EH, Watson DMS (1914). On the flight of pterodactyls.. Aeronautical Journal.

[114] Frey E, Buchy MC, Martill DM, Buffetaut E, Mazin JM (2003). Middle- and bottom- decker Cretaceous pterosaurs: unique designs in active flying vertebrates.. Geological Society Special Publication.

[115] Elgin RA, Hone DWE, Frey E (In press). The extent of the pterosaur flight membrane..

[116] Brower JC (1983). The aerodynamics of *Pteranodon* and *Nyctosaurus*, two large pterosaurs from the upper Cretaceous of Kansas.. Journal of Vertebrate Paleontology.

[117] Bennett SC (2005). [Combined book review of] Posture, Locomotion, and Paleoecology of Pterosaurs by S. Chatterjee and R. J. Templin and Evolution and Palaeobiology of Pterosaurs edited by E. Buffetaut and J.-M. Mazin.. Journal of Paleontology.

[118] Alexander D (2002). Nature's Flyers: Birds, Insects, and the Biomechanics of Flight.

[119] Campbell KE, Tonni EP (1983). Size and locomotion in teratorns (Aves: Teratornithidae).. The Auk.

[120] Mayr GA, Hazevoet CJ, Dantas P, Cachão M (2008). Sternum of a Very Large Bony-Toothed Bird (Pelagornithidae) from the Miocene of Portugal.. Journal of Vertebrate Paleontology.

[121] Witton MP, Martill DM, Green M (2009). On pterodactyloid diversity in the British Wealden (Lower Cretaceous) and a reappraisal of *“Palaeornis” cliftii* Mantell, 1844.. Cretaceous Research.

[122] Savile DBO (1957). Adaptive evolution of the avian wing.. Evolution.

[123] Hertel F, Balance LT (1999). Wing ecomorphology of seabirds from Johnston Atoll.. Condor.

